# Trimacrocyclic hexasubstituted benzene linked by labile octahedral [X(CHCl_3_)_6_]^−^ clusters†

**DOI:** 10.1039/d1sc03713g

**Published:** 2021-08-12

**Authors:** Zhenzhen Lai, Aimin Li, Sangshan Peng, Jonathan L. Sessler, Qing He

**Affiliations:** State Key Laboratory of Chemo/Biosensing and Chemometrics, College of Chemistry and Chemical Engineering, Hunan University Changsha 410082 P. R. China heqing85@hnu.edu.cn; Department of Chemistry, The University of Texas at Austin 105 East 24^th^ Street, Stop A5300 Austin Texas 78712 USA

## Abstract

Crystalline supramolecular architectures mediated by cations, anions, ion pairs or neutral guest species are well established. However, the robust crystallization of a well-designed receptor mediated by labile anionic solvate clusters remains unexplored. Herein, we describe the synthesis and crystalline behaviors of a trimacrocyclic hexasubstituted benzene **2** in the presence of guanidium halide salts and chloroform. Halide hexasolvate clusters, *viz.* [Cl(CHCl_3_)_6_]^−^, [Br(CHCl_3_)_6_]^−^, and [I(CHCl_3_)_6_]^−^, were found to be critical to the crystallization process, as suggested by the single-crystal structures, X-ray powder diffraction (XRPD), thermogravimetric analysis (TGA), scanning electron microscopy with energy dispersive spectroscopy (SEM-EDS), and NMR spectroscopy. This study demonstrates the hitherto unexpected role that labile ionic solvate clusters can play in stabilizing supramolecular architectures.

## Abstract 2

We report the synthesis and robust crystallization of a trimacrocyclic hexasubstituted benzene and guanidium mediated by unprecedented labile halide hexasolvate clusters, *viz.* [Cl(CHCl_3_)_6_]^−^, [Br(CHCl_3_)_6_]^−^, [I(CHCl_3_)_6_]^−^, and [Br(CHBr_3_)_6_]^−^.

## Graphical abstract

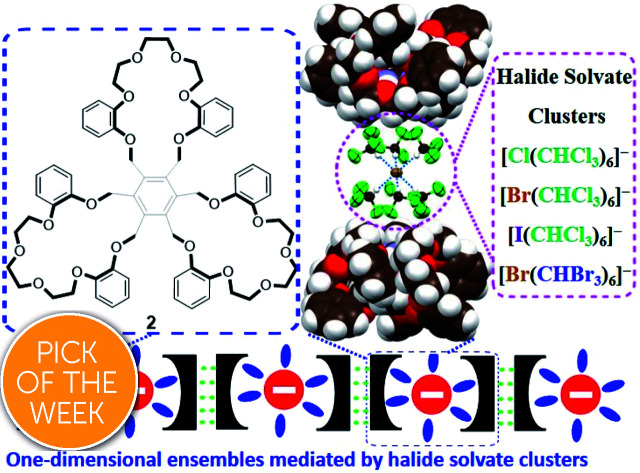

## Introduction

**Fig. 1:**
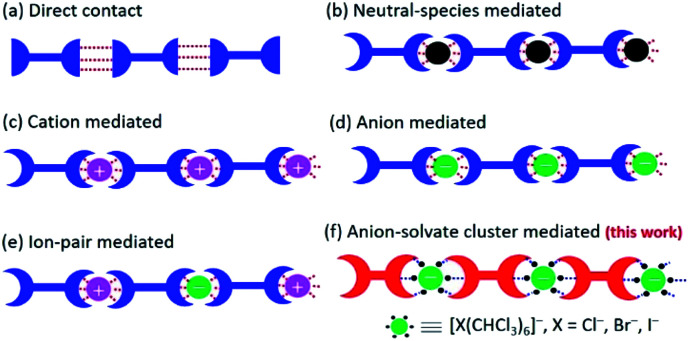
Cartoon illustration of supramolecular systems stabilized by (a) direct contact or mediated by intervening (b) neutral guests, (c) cations, (d) anions, (e) ion-pairs, or (f) octahedral anion solvate clusters (this work). Noncovalent interactions are indicated by red or blue dotted lines.

Crystallization or co-crystallization is ubiquitous in biology, chemistry, materials science, and manufacturing.^1^ It offers a very powerful synthetic strategy for fabricating interesting and useful ordered ensembles of molecules, *e.g.*, nanoparticles, molecular crystals, colloids, semiconductor quantum dots, and phase-separated polymers.^2–7^ A particular subset of crystallization involves the formation of ensembles stabilized by non-covalent interactions. The resulting systems can be classified generally as those stabilized by (a) direct contact, wherein the individual components are connected through supramolecular interactions^8^ or mediated by intervening (b) neutral guests,^9^ (c) cations,^10^ (d) anions,^11–13^ or (e) ion-pairs (Fig. 1).^14^ Currently, we are unaware of any well-defined supramolecular constructs whose formation is mediated by labile anion solvate clusters, such as [X(CHCl_3_)_6_]^−^ (X = Cl^−^, Br^−^, I^−^). Here we report what to the best of our knowledge are the first examples wherein [Cl(CHCl_3_)_6_]^−^, [Br(CHCl_3_)_6_]^−^, [I(CHCl_3_)_6_]^−^ and [Br(CHBr_3_)_6_]^−^ serve to promote the solid state crystallization of a trimacrocyclic hexasubstituted benzene (THB) **2** as inferred from single crystal X-ray diffraction analyses. Under conditions of rapid hexanes-induced precipitation, highly ordered cubic particles are formed.

Chloroform (CHCl_3_) is a common organic solvent, widely used both in the laboratory and in industry. Chloroform can exert a pronounced solvent effect, particularly in the presence of halide anions.^15–20^ Halide solvate clusters, such as [X(CHCl_3_)_*n*_]^−^ where X = Cl^−^, Br^−^, I^−^, have attracted interest as models with which to explore these effects.^21–27^ On account of the weak X^−^⋯H–C interaction between the central halide anion and the chloroform molecules, [X(CHCl_3_)_*n*_]^−^ clusters are inherently labile. As a result, most work to date has focused on theoretical investigations, as well as mass spectrometric and single-crystal X-ray crystallographic studies of clusters with low coordination numbers (*n* < 4).^21,23,25^ Although a few examples have been reported of [X(CHCl_3_)_*n*_]^−^ clusters with high coordination number (*e.g.*, [Cl(CHCl_3_)_6_]^−^), in certain lattices in the presence of transition metals.^28,29^ We are unaware of examples where [X(CHCl_3_)_*n*_]^−^ clusters drive crystallization. As detailed below, we have now found that [X(CHCl_3_)_6_]^−^ clusters can drive formation of well-organized, non-covalent crystalline structures.

## Results and discussion

### Design, synthesis and characterization of receptor **2**

**Fig. 2:**
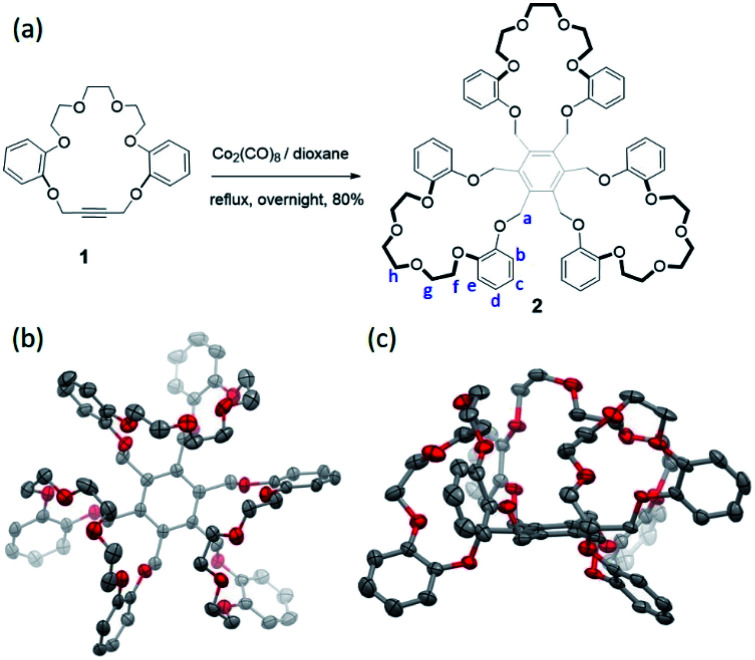
(a) Co_2_(CO)_8_-catalyzed synthesis of receptor **2**; (b) top view and (c) front view of the solid-state structure of **2** as determined *via* single-crystal X-ray diffraction analysis. All solvent molecules and hydrogen atoms are omitted for clarity.

Hexasubstituted benzenes represent a versatile class of frameworks for constructing supramolecular receptors for either anions or cations.^30–33^ Usually, such architectures can access a variety of conformations due to the high degree of freedom of the substituents on the benzene core; this makes host–guest interactions with a putative substrate less entropically favorable. One way to overcome this latter energic penalty is to reduce the degrees of freedom by means of macrocyclization so as to generate, *e.g.*, trimacrocyclic hexasubstituted benzene (THB) derivatives.^33,34^ Taking advantage of this latter strategy we have now prepared a trimacrocyclic hexasubstituted benzene **2** receptor wherein crown ethers are incorporated into the system (Fig. 2a). The synthesis of **2** is straightforward. Briefly, a key crown ether-like precursor **1** containing acetylene unit was prepared according to a reported approach (see Scheme S1†).^35^ A Co_2_(CO)_8_-catalyzed trimerization of **1** in dioxane at reflux overnight then gave receptor **2** in 80% yield after chromatographic purification over silica gel. The identity of **2** was confirmed by ^1^H/^13^C NMR spectroscopic and high-resolution mass spectrometric analyses (see the ESI†).

Diffraction grade single crystals of receptor **2** were obtained by allowing a CHCl_3_/CH_3_OH solution of **2** to undergo slow evaporation over the course of two weeks. The resulting structure revealed a *cis*-like conformation, where all three crown ether moieties are oriented toward the same face of the benzene core (Fig. 2b and see ESI Fig. S1†). This finding led us to postulate that **2** might serve as a receptor for guanidium cations by virtue of its size, symmetry and presumed shape complementary (Fig. S2†).

### Host–guest binding properties of receptor **2**

To test the hypothesis that compound **2** could serve as a receptor for guanidium cation, we carried out ^1^H NMR spectroscopic experiments with receptor **2** and guanidium chloride in a mixture of CDCl_3_/CD_3_OD (9 : 1, v/v) at 298 K. Notably, only one set of resonances was seen for **2** under these conditions (Fig. S3†); this is as expected for a relatively flexible system that is in conformational equilibrium. Upon addition of excess guanidium chloride into a 2.0 mM solution of **2** in CDCl_3_/CD_3_OD (9 : 1, v/v), the C_sp^3^_–H peaks at 5.43 ppm (*a*), 3.94 ppm (*f*) were shifted upfield (Fig. S3†). In contrast, negligible changes were observed for any of the proton signals upon the addition of other larger and unsymmetrical guanidium chloride derivatives, *viz.* moroxydine hydrochloride (**S6**), and 1,1-dimethylbiguanide hydrochloride (**S7**), under identical conditions. Thus, we infer that receptor **2** favors guanidium chloride over other potential competing substrates. A ^1^H NMR spectroscopic titration yielded an association constant of *K* = (1.6 ± 0.4) × 10^4^ M^−1^ for the interaction of guanidium chloride with **2** in a 1 : 1 binding model (Fig. S4 and S5†). The stability of the resulting complex was also evidenced by gas-phase molecular dynamics simulation studies (Fig. S6†).

### [X(CHCl_3_)_6_]^−^ cluster-mediated single crystallization

**Fig. 3:**
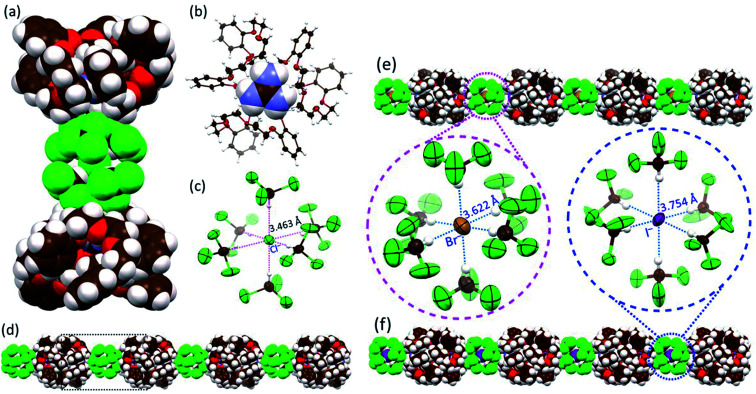
Different views and packing modes of single-crystal structures. (a) Complex **2**·CN_3_H_6_^+^·CN_3_H_5_·[Cl(CHCl_3_)_6_]^−^ shown in space-filling form; (b) host–guest complex **2**·0.5CN_3_H_6_^+^·0.5CN_3_H_5_ with the receptor **2** shown in an ellipsoid model with the guanidium guest shown in space-filling form; (c) a [Cl(CHCl_3_)_6_]^−^ cluster within the overall structure with hydrogen bonds indicated by red dotted lines for which a uniform Cl^−^⋯C(HCl_3_) distance of 3.463 Å is seen; views of the highly ordered linear architectures formed by dimeric **2**_2_·CN_3_H_6_^+^·CN_3_H_5_ complexes and (d) [Cl(CHCl_3_)_6_]^−^ clusters; (e) [Br(CHCl_3_)_6_]^−^ clusters; and (f) [I(CHCl_3_)_6_]^−^ clusters shown in space-filling form. In (e and f), also shown are the [Br(CHCl_3_)_6_]^−^ and [I(CHCl_3_)_6_]^−^ clusters displayed as ellipsoid models with hydrogen bonds indicated by blue dotted lines.

Further evidence that compound **2** could act as a cation receptor for guanidium came from a single-crystal X-ray diffraction analysis of the guanidium chloride complex. Suitable crystals were obtained *via* the slow evaporation of a CHCl_3_/CH_3_OH solution of receptor **2** in the presence of excess guanidium chloride. The resulting structure revealed a **2**_2_·CN_3_H_6_^+^·CN_3_H_5_·[Cl(CHCl_3_)_6_]^−^ complex (Fig. 3a). Due to the limitations of crystallography, positively charged guanidium and the charge-free guanidine species could not be distinguished from one another. From the relative number of chloride anions, we infer that the occupancies of guanidium and guanidine in each cavity of **2** are each 50%. As expected, the guest species was embedded within the cavity of **2** surrounded by three cyclic glycol chains *via* multiple C–H⋯O, N–H⋯O hydrogen bonding and cation-π interactions (Fig. 3b and S7†). A dimeric complex **2**_2_·CN_3_H_6_^+^·CN_3_H_5_ is found in the lattice (Fig. S8†). Much to our surprise, a careful inspection of the counter anions revealed that each chloride anion was surrounded by six chloroform molecules through multiple hydrogen bonding interactions within a [Cl(CHCl_3_)_6_]^−^ cluster characterized by an average Cl^−^⋯C(HCl_3_) distance of 3.463 Å (Fig. 3c). Although [Cl(CHCl_3_)_*n*_]^−^ clusters have been reported to be labile due to the weak Cl^−^⋯H–C(HCl_3_) bonds,^23^ in the current system, the octahedral [Cl(CHCl_3_)_6_]^−^ clusters are found to mediate the formation of highly ordered 1D (one dimensional) supramolecular architectures (Fig. 3d) that are arranged in 2D networks (Fig. S8 and S9†).

We next sought to explore whether other similar octahedral halide chloroform clusters, *i.e.*, [Br(CHCl_3_)_6_]^−^ and [I(CHCl_3_)_6_]^−^, could be stabilized in the solid state and mediate the generation of analogous supramolecular assembles as seen for the chloride anion. As above, diffraction-grade single crystals were obtained by slowly evaporating CHCl_3_/CH_3_OH solutions of **2** in the presence of guanidium bromide and guanidium iodide, respectively. The resulting structures revealed that, in analogy with what was seen in the presence of guanidium chloride, in both **2**_2_·CN_3_H_6_^+^·CN_3_H_5_·[Br(CHCl_3_)_6_]^−^ (the complex formed from guanidium bromide) and **2**_2_·CN_3_H_6_^+^·CN_3_H_5_·[I(CHCl_3_)_6_]^−^ (the complex formed from guanidium iodide) cluster-linked ensembles were observed (Fig. 3e and f and ESI Fig. S10 to S17†). Again, both bromide and iodide were found surrounded by six chloroform molecules with average anion–solvent distances of 3.622 Å and 3.754 Å for Br^−^⋯C(HCl_3_) and I^−^⋯C(HCl_3_), respectively, being found (Fig. 3e and f). The [Br(CHCl_3_)_6_]^−^ and [I(CHCl_3_)_6_]^−^ clusters were observed to bridge the individual **2**_2_·CN_3_H_6_^+^·CN_3_H_5_·[X(CHCl_3_)_6_]^−^ complexes, resulting in the formation of 1D assemblies and 2D networks (Fig. 3e and f, see ESI Fig. S13 and S17†). Interestingly, upon replacement of CHCl_3_ with CHBr_3_, an analogous crystal structure of **2**_2_·CN_3_H_6_^+^·CN_3_H_5_·[Br(CHBr_3_)_6_]^−^ complex was obtained in success and similar 1D assemblies and 2D networks were observed (Fig. S18 to S21†).

### [X(CHCl_3_)_6_]^−^ cluster-mediated rapid crystallization experiments carried out under more general conditions

**Fig. 4:**
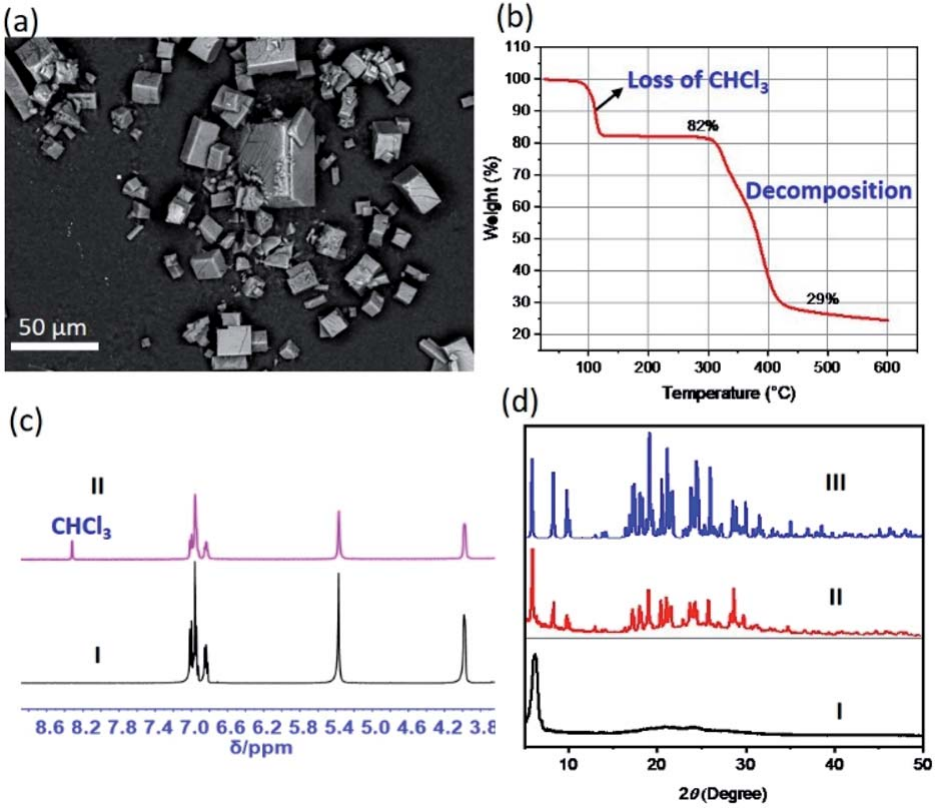
Characterization of [Cl(CHCl_3_)_6_]^−^ cluster-mediated crystalline constructs under more general conditions. (a) SEM micrograph of cubic microcrystalline particles obtained by mixing **2** with guanidium chloride in the presence of CHCl_3_ under conditions of rapid precipitation (see text for details); (b) thermogravimetric decomposition trace for the particles in (a); (c) selected regions of the ^1^H NMR spectra of solutions of receptor **2** in DMSO-d_6_ (I) and the particles from (a) redissolved in DMSO-d_6_ (II); and (d) PXRD patterns of (I) solid obtained by drying **2** in the presence of CHCl_3_ but in the absence of a guest, (II) particles shown in (a), and (III) simulated PXRD pattern using the data from the single-crystal structure of complex **2**·CN_3_H_6_^+^·CN_3_H_5_·[Cl(CHCl_3_)_6_]^−^.

All single crystals giving rise to the structurally characterized **2**_2_·CN_3_H_6_^+^·CN_3_H_5_·[X(CHCl_3_)_6_]^−^ complexes (X = Cl^−^, Br^−^, I^−^) were grown over a period of several weeks. We were thus curious to explore whether labile [X(CHCl_3_)_6_]^−^ clusters would support the robust co-crystallization of receptor **2** and guanidium halide in the presence of chloroform under more general conditions, such as those associated with rapid mixing and precipitation. To test this hypothesis, we sparged hexanes into a CHCl_3_/CH_3_OH (2 : 1, v/v) solution of **2** in the presence of **1** molar equivalent of guanidium chloride. This led to near immediate precipitation of what were found to be cubic crystalline particles as viewed under a polarizing microscope (Fig. S22 to S24†). These morphological features were further characterized by scanning electron microscopy (SEM) (Fig. 4a and ESI Fig. S25†). To gain greater insight into the nature of the cube-shaped microcrystalline material, SEM-EDS (energy dispersive spectrometry) experiments were performed. EDS elemental mapping associated with an SEM image revealed the occurrence of the expected elements (*i.e.*, C, N, O, and Cl). This was taken as evidence that the microcrystals consisted of, at least, receptor **2** and guanidium chloride (Fig. S26†).

Thermogravimetric analyses (TGA), ^1^H NMR spectroscopic studies, and powder X-ray diffraction (PXRD) measurements were then carried out in an effort to confirm the inference that the microcrystalline material contained [Cl(CHCl_3_)_6_]^−^ clusters. The above-mentioned precipitates were filtered off and dried naturally in a fume hood to allow evaporation of potential solvent residues on the surfaces. The resulting samples were subjected to TGA, NMR spectroscopic and PXRD analyses. Briefly, the TGA trace shows an initial mass loss of approximately 20% of the initial sample weight near 100 °C, a finding consistent with the release of CHCl_3_ from the crystalline materials (Fig. 4b). Additionally, a sharp CHCl_3_ peak was observed in the ^1^H NMR spectrum acquired by redissolving the microcrystals in DMSO-*d*_6_ (Fig. 4c). The observed content of CHCl_3_ in the precipitates were found slightly lower than that (22%) seen in the corresponding single crystal structures, which could be rationalized by the loss of CHCl_3_ during the sample preparation, drying and transfer due to the labile nature of the clusters (Fig. S27†). Finally, the PXRD pattern of the crystalline material was fully in accord with the simulated PXRD pattern using the diffraction data for the **2**·CN_3_H_6_^+^·CN_3_H_5_·[Cl(CHCl_3_)_6_]^−^ single crystals (Fig. 4d and S28†). In contrast, when chloroform was replaced by dichloromethane in the mixing of **2** with guanidium chloride, only column-like crystalline ensembles of **2** without guest species were observed (Fig. S29 and S30†). Taken together, these findings provide support for the notion that the acidic C–Hs of the CHCl_3_ serve as hydrogen bonding donors and play an essential role in holding together six chloroform molecules as labile octahedral halide anion solvate clusters (such as [Cl(CHCl_3_)_6_]^−^) that mediate formally the co-crystallization of **2** and guanidium chloride.

When mixtures of receptor **2** and guanidium bromide or guanidium iodide (in 1 : 1 ratios) were allowed to co-crystalize under otherwise identical conditions, cubic crystalline entities were also observed; again, the resulting constructs were fully characterized by microscopic techniques (Fig. S31–S33, S37 and S38†). The fact that [Br(CHCl_3_)_6_]^−^ and [I(CHCl_3_)_6_]^−^ clusters support the formation of cubic microcrystal materials was inferred from SEM-EDS experiments, thermogravimetric analysis, ^1^H NMR spectroscopy and powder X-ray diffraction studies as used for the [Cl(CHCl_3_)_6_]^−^ cluster species described above (Fig. S33–S36 and S38–S41†).

## Conclusions

In summary, a trimacrocyclic hexasubstituted benzene derivative **2** was synthesized *via* a Co_2_(CO)_8_-catalyzed [2 + 2 + 2] tricyclization of a monoyne-containing crown ether. This 3-fold symmetric system was found capable of trapping guanidium in a 1 : 1 ratio with an association constant of *K* = (1.6 ± 0.4) × 10^4^ M^−1^ in a mixture of chloroform and methanol (9 : 1, v/v). A series of [X(CHCl_3_)_6_]^−^ clusters, *viz.* [Cl(CHCl_3_)_6_]^−^, [Br(CHCl_3_)_6_]^−^, [I(CHCl_3_)_6_]^−^, and [Br(CHBr_3_)_6_]^−^, were observed to mediate the formation of 1D and 2D supramolecular entities as reflected in the corresponding single crystal X-ray diffraction-based structures. The octahedral halide chloroform clusters seen in the solid state proved key to the formation of cubic crystalline entities under conditions of rapid mixing and precipitation. To the best of our knowledge, this study represents the first example wherein labile halide solvate clusters serve to promote co-crystallization in the solid state. It may thus help advance our understanding how solvents affect organization at the molecular level while illustrating a new recognition motif that could prove useful in the creation of yet-more elaborate supramolecular architectures.

## Data availability

All associated experimental and computational details are provided in the ESI.†

## Author contributions

Conceptualization and supervision: QH; synthesis, characterization, NMR, XRD, and TGA studies: ZL; single crystal growing, data collection and analysis: ZL and QH; theoretical calculations: AL; SEM-EDS experiments: SP; writing – original draft: ZL and QH; writing – review & editing, QH and JLS. All authors proofread, commented on, and approved the final version of this manuscript.

## Conflicts of interest

There are no conflicts to declare.

## Supplementary Material

SC-012-D1SC03713G-s001

SC-012-D1SC03713G-s002
