# Supplementary material for: Trimacrocyclic hexasubstituted benzene linked by labile octahedral [X(CHCl3)6]− clusters
Source: Chem Sci. 2021 Aug 12;12(35):11647–51. doi: 10.1039/d1sc03713g (PMC8442620; doi:10.1039/d1sc03713g)
Supplement: SC-012-D1SC03713G-s001 [file SC-012-D1SC03713G-s001.pdf]

Electronic Supplementary Material (ESI) for Chemical Science.
This journal is © The Royal Society of Chemistry 2021

Supporting Information

For

Trimacrocyclic hexasubstituted benzene linked by labile octahedral $[X(CHCl_3)_6]^-$ clusters

Zhenzhen Lai,† Aimin Li,† Sangshan Peng,† Jonathan L. Sessler,‡ and Qing He*,†

† State Key Laboratory of Chemo/Biosensing and Chemometrics, College of Chemistry and Chemical Engineering, Hunan University, Changsha 410082, P. R. China.

‡ Department of Chemistry, The University of Texas at Austin, Austin, Texas 78712-1224, United States.

* Corresponding author: Qing He (E-mail: heqing85@hnu.edu.cn).

Contents

1. General information
2. Synthesis
3. Crystal structures, calculations and binding studies
4. Rapid crystallization experimental
5. X-ray experimental details
6. HRMS spectra and NMR spectra
7. References

1. General information

All solvents and chemicals used were purchased from Sigma–Aldrich, TCI, Energy–Chemical, or Acros and used without further purification. TLC analyses were carried out using Sorbent Technologies silica gel (200 mesh) sheets. Flash column chromatography was performed on silica gel (300–400 mesh). ^{1}H and ^{13}C NMR spectra were recorded on Bruker AVANCE 400 spectrometers and the spectroscopic solvents were purchased from Cambridge Isotope Laboratories or Sigma–Aldrich. Tetramethylsilane (TMS) was used as an internal reference. The chemical shifts are expressed in δ (ppm). High–resolution mass spectra (HRMS) were recorded on a Bruker Apex–Q IV FTMS mass spectrometer using ESI (electrospray ionization) employing a mixture of $CHCl_3/CH_3OH$ (9:1, v/v) as the solvent. X–ray crystallographic analyses were carried out on a Brucker D8 Advance diffractometer using a *μ*–focused Mo Kα radiation source (λ = 0.71073 Å) an Agilent Technologies SuperNova Dual Source diffractometer using a μ-focused Cu Kα radiation source (λ = 1.5418 Å) with collimating mirror monochromators. Scanning Electron Microscope (SEM) images were acquired by using MIRA3 LMH (TESCAN) microscope and the corresponding energy dispersive spectroscopy (EDS) mapping was obtained by an EDS detector (X MAX20, Oxford). Thermo-gravimetric analysis (TGA, HCT-1) was measured under argon atmosphere with a heating rate of 10 °C min^{-1}. X-ray diffraction (XRD) patterns were collected by D/Max 2500VB using Cu *Kα* radiation. All theoretical calculations were carried out with the Gaussian 09 suite[1] of programs using the X3LYP density functional.[2] Structural optimization was performed using a 6–31G* basis set while single–point energies were calculated using the 6–31+g* basis set. Complexation energies were corrected for basis set superposition error (BSSE) using the counterpoise correction method.[3-4]

2. Synthesis

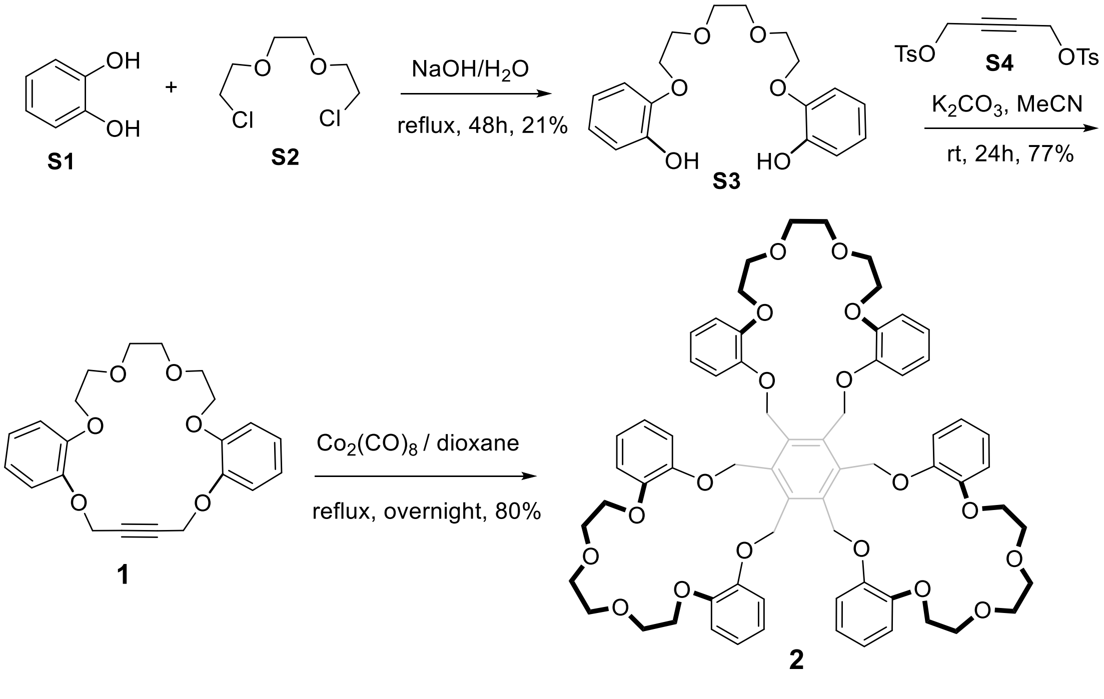

Scheme S1. Synthesis of receptor **2**.

Compounds **S1** and **S2** are commercially available.

Synthesis of S3

To a stirred refluxing solution of catechol (27.60 g, 250.0 mmol) and NaOH (3.46 g, 86 mmol) dissolved in water (200 mL) under nitrogen was added dropwise triethylene glycol dichloride (7.86 g, 42.0 mmol) for 3 h. The mixture was heated at reflux for 48 h and cooled to room temperature. It was then extracted with EtOAc and the organic phases combined. The organic phase was dried over anhydrous MgSO4, filtered, and then concentrated under reduced pressure. The resulting crude product was subjected to column chromatography over silica gel (petroleum ether /ethyl acetate = 2:1, eluent) to afford solid **4** as a white solid (3.00 g, 21% yield). 1H NMR (400 MHz, DMSO-d_6) δ 8.87 (s, 2H), 6.91 (d, *J* = 7.8 Hz, 2H), 6.82-6.68 (m, 6H), 4.06 (t, *J* = 4.7 Hz, 4H), 3.75 (t, *J* = 4.8 Hz, 4H), 3.63 (s, 4H). ^{13}C NMR (100 MHz, DMSO-d_6) δ 147.4, 147.2, 121.8, 119.7, 116.2, 114.7, 70.4, 69.5, 68.6.[5]

Synthesis of S4

2-Butyne-1,4-diol (4.30 g, 50.0 mmol) was dissolved in THF (100 mL) and mixed with NaOH (7.00 g, 175.0 mmol) dissolved in water (100 mL). After cooling to 0 °C, a solution of *p*-tosyl chloride (20.97 g, 110.0 mmol) in THF (150 mL) was added dropwise to this mixture over the course of 1 h. The mixture was stirred at 0 °C for 2 h and then extracted with EtOAc (2 x 250 mL) and washed with saturated $NaHCO_3$ and brine in succession. The organic layer was dried over anhydrous MgSO4, filtered and then concentrated under reduced pressure to give a grey solid (17.8 g, 90% yield). ^{1}H NMR (400 MHz, $CDCl_3$) δ 7.78-7.74 (d, 4H), 7.37-7.33 (d, 4H), 4.58 (s, 4H), 2.45 (s, 6H). ^{13}C NMR (100 MHz, $CDCl_3$) δ 145.5, 132.8, 130.0, 128.1, 81.0, 57.1, 21.7.[6]

Synthesis of precursor 1

To a stirred solution of bisphenol **S3** (575 mg, 1.72 mmol) in dry MeCN (20 mL) was added K_2CO_3 (1.18 g, 8.60 mmol). The reaction mixture was then heated at reflux for 30 min. A dry MeCN (20 mL) solution of 2-butyne-1,4-diol ditosylate **S4** (678 mg, 1.72 mmol) was then added to the mixed solution, which was heated at reflux for 24 h. The reaction mixture was cooled and filtered. The filtrate was concentrated under reduced pressure to give the crude product, which was further purified by silica gel column chromatography (ethyl acetate/petroleum ether = 2:1, eluent) to afford **1** as a white solid (509 mg, 77% yield). ^{1}H NMR (400 MHz, $CDCl_3$) δ 6.98-6.93 (m, 2H), 6.92-6.86 (m, 6H), 4.74 (s, 4H), 4.17 (dd, *J* = 5.9, 2.8 Hz, 4H), 3.95-3.89 (m, 8H). ^{13}C NMR (100 MHz, $CDCl_3$) δ 149.4, 147.6, 122.6, 121.4, 115.5, 114.3, 82.0, 71.5, 70.0, 69.3, 57.8. HRMS (ESI) m/z 407.1465 $[M + Na]^+$ calcd for $C_{22}H_{24}NaO_6$, found 407.1460.[7]

Synthesis of compound 2

To a solution of intermediate **1** (3.23 g, 2.9 mmol) in anhydrous dioxane (50 mL) was added $Co_2(CO)_8$ (100 mg, 0.29mmol) in an oven dried Schlenk flask. The reaction mixture was heated at reflux for 16 h under an N_2 atmosphere. At the conclusion of the reaction (as inferred from TLC monitoring), the filtrate was concentrated and further purified by silica gel column chromatography (dichlormethane/methanol = 20:1, eluent) to afford **2** (892 mg, 80% yield) as a white solid. ^{1}H NMR (400 MHz, $CDCl_3$) δ 7.00 (dd, *J* = 8.2, 1.6 Hz, 5H), 6.96-6.91 (m, 6H), 6.87-6.81 (m, 12H), 5.52 (s,

12H), 4.05-3.99 (m, 12H), 3.53 (d, $J = 10.0$ Hz, 24H). ^{13}C NMR (100 MHz, $CDCl_3$) δ 150.4, 148.3, 138.2, 122.5, 121.0, 117.6, 113.0, 71.7, 69.3, 68.9, 66.0. HRMS (ESI) m/z 1175.4611 $[M + Na]^+$ calcd for $C_{74}H_{85}N_{14}O_4$, found 1175.4611.

3. Crystal structures and binding studies

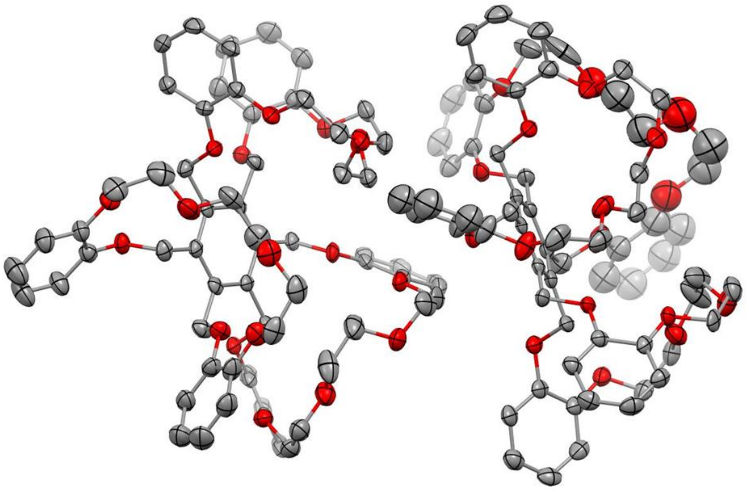

Fig. S1. The X-ray diffraction structure of compound **2** shown in ellipsoid model form obtained from single crystals grown by subjecting a solution of **2** in $CHCl_3$/methanol to slow evaporation. In each asymmetrical unit cell, two molecules of **2** are observed, both of which adopt almost the same *cis* conformation. The molecule on the left was selected and shown in Fig. 1 of the main text. Displacement ellipsoids are scaled to the 50% probability level. Many solvent molecules were found to be disordered and removed with the SQUEEZE procedure in PLATON.[8] All hydrogen atoms are omitted for clarity.

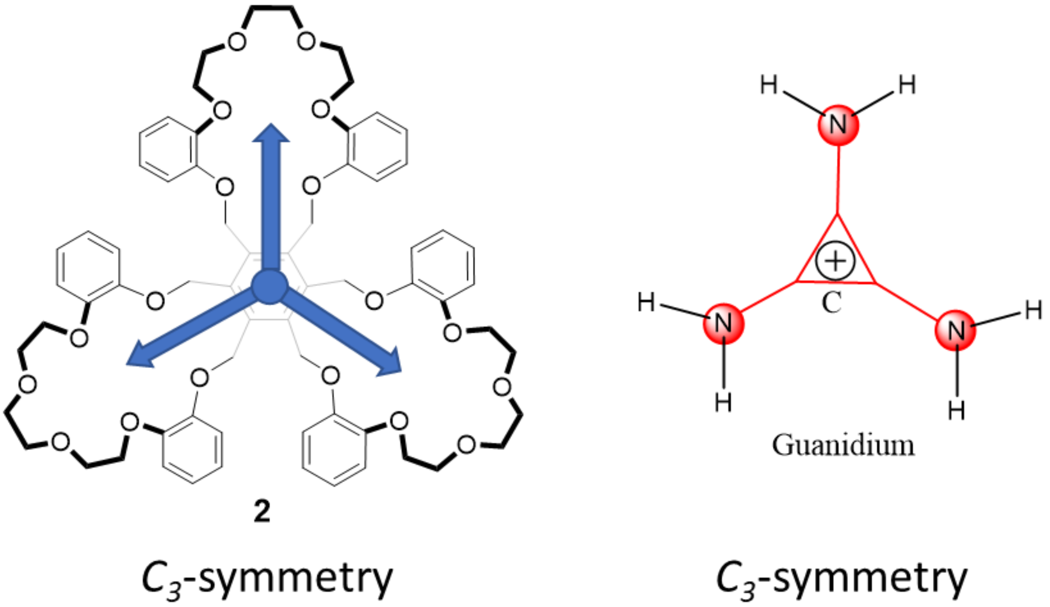

Fig. S2. Symmetry analysis of receptor **2** and the putative cationic guest, guanidium. Both receptor **2** and guanidium possess C_3 symmetry but are characterized by the presence of hydrogen bonding acceptors and donors, respectively.

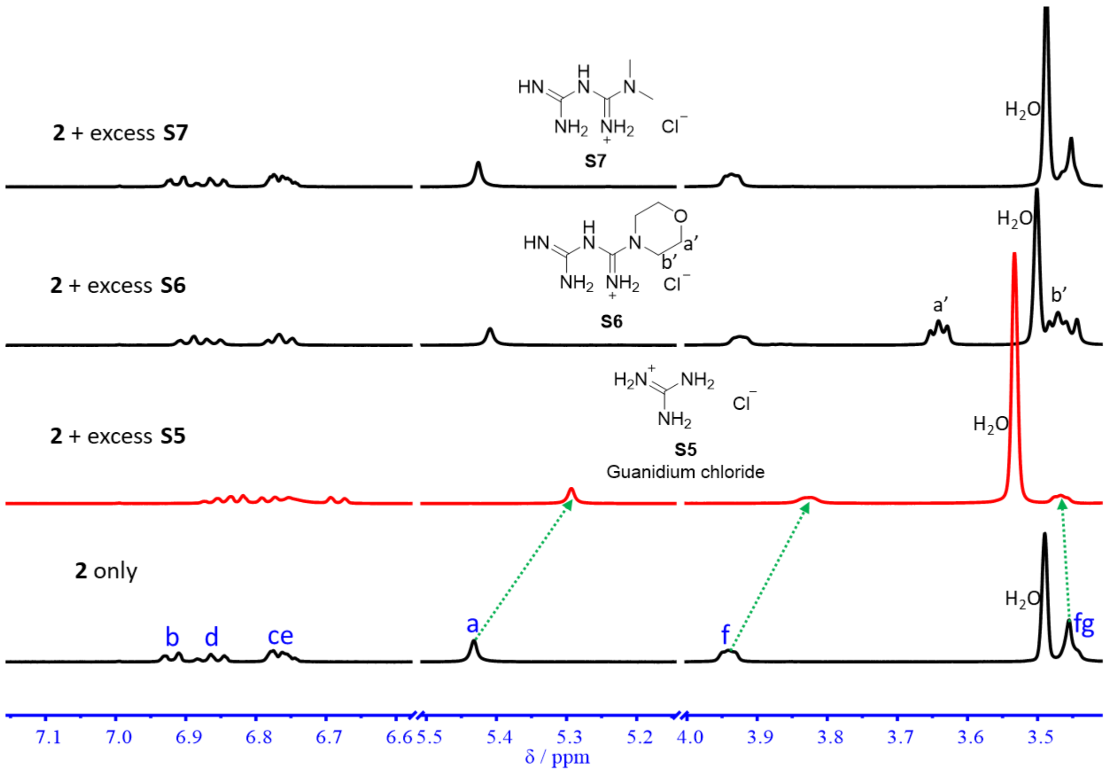

Fig. S3. Selected regions of the ^{1}H NMR spectra ($CDCl_3/CD_3OD$ (9:1, v/v), 298 K) of solutions of **2** recorded in the absence or presence of excess guanidium chloride (**S5**), moroxydine hydrochloride (**S6**), and 1,1-dimethylbiguanide hydrochloride (**S7**), respectively.

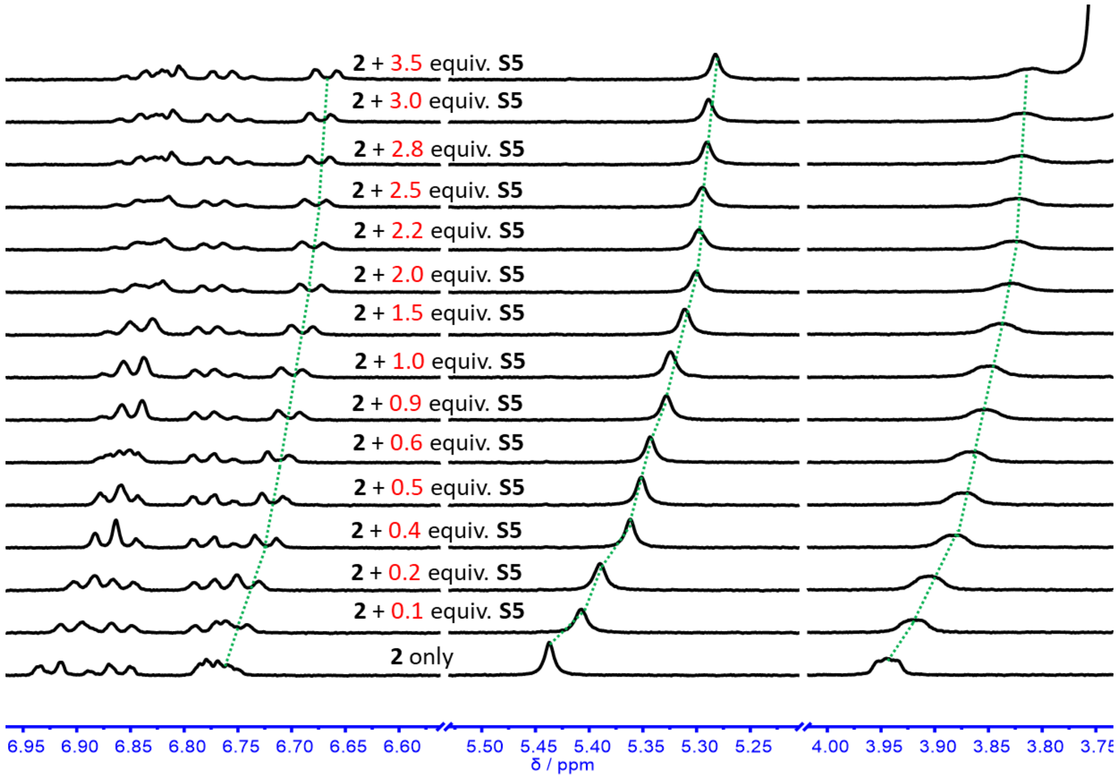

Fig. S4. Selected regions of ^{1}H NMR spectra ($CDCl_3$/CD_3OD (9:1, v/v), 298 K) acquired during the titration of **2** with increasing quantities of guanidium chloride (**S5**): 0, 0.05, 0.1, 0.2, 0.3, 0.4, 0.5, 0.6, 0.9, 1.0, 1.5, 2.0, 2.2, 2.8, 3.0, and 3.5 equiv. The dashed lines have been added to aid in visualization.

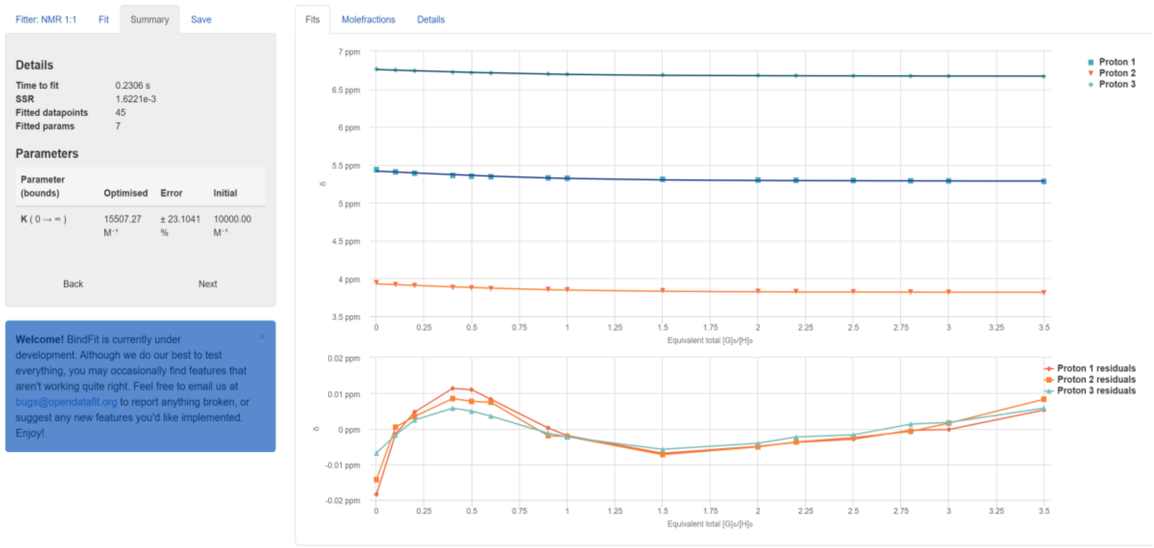

Fig. S5. Nonlinear least-square analysis of the ^{1}H NMR binding data corresponding to the formation of complex [**2•S5**]$^+$. The data were fitted to a 1:1 binding model to give K = (1.6 ± 0.4) ×10^4 M^{-1}. The residual distribution is shown below the binding isotherm. All solid lines were obtained from non-linear curve-fitting to a 1:1 binding model using the www.supramolecular.org web applet.[9]

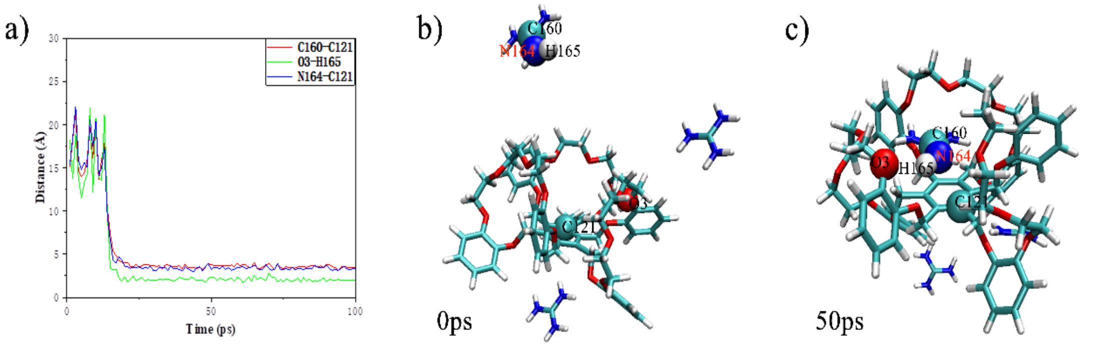

Fig. S6. Molecular dynamics run on a system comprising one equiv of host **2** and three guanidine groups in the gas phase using periodic boundary conditions at 300 K. (a) Traces of selected distances versus time; the key elements, e.g. O3, C121, C160, N164 and H165, are labeled in the molecular models; structures at 0 ps (b) and 50 ps (c). Host **2** captures the guanidine guest **S5** after 20 ps. Three guanidium molecules are randomly distributed around one receptor **2** but only one guanidium was found to be trapped by **2** and the resulting complex proved very stable under the conditions of these calculations.

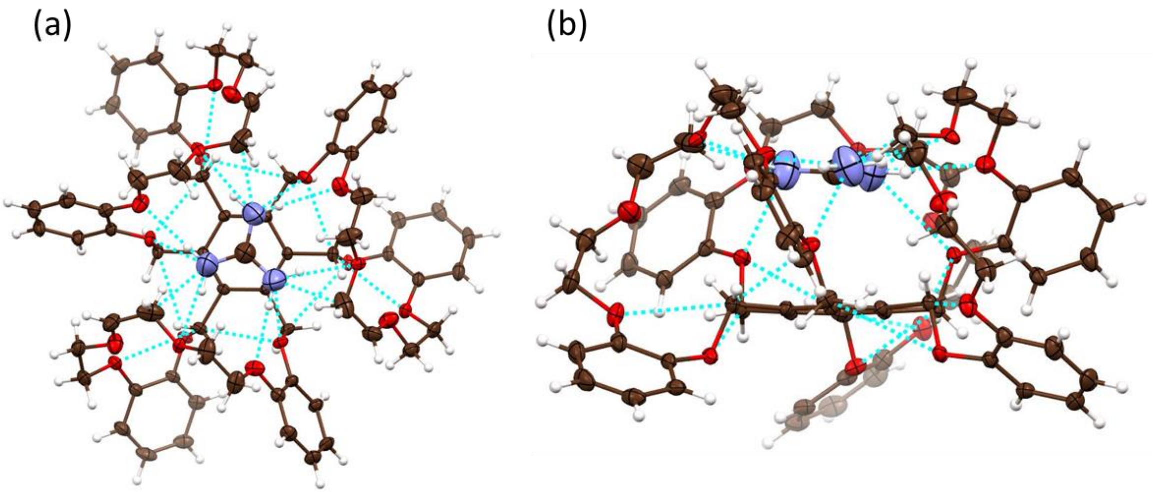

Fig. S7. (a) Top and (b) front views of the crystal structure of complex **2**·0.5$CN_3H_6^+$·0.5CN_3H_5 shown in ellipsoid form. The single crystals used for this analysis were grown by subjecting a solution of **2** in $CHCl_3$/methanol to slow evaporation in the presence of excess guanidium chloride. Displacement ellipsoids are scaled to the 50% probability level. Inferred hydrogen bonds are indicated by blue dotted lines.

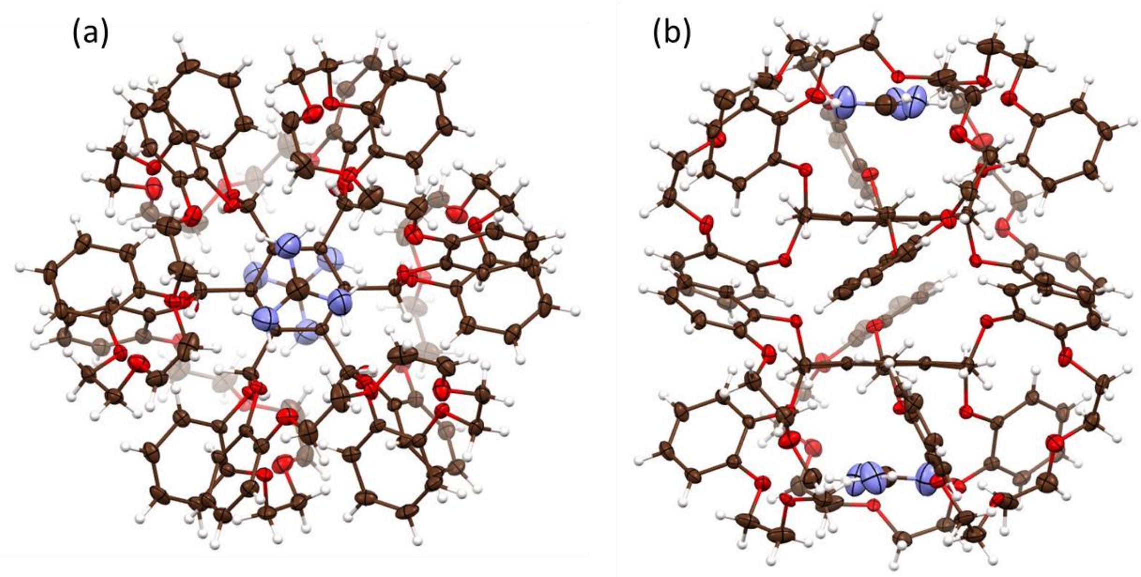

Fig. S8. (a) Top and (b) front views of the dimeric complex $\mathbf{2}_2 \cdot CN_3H_6^+ \cdot CN_3H_5$ shown in ellipsoid form. Single crystals used for this analysis were grown by subjecting a solution of **2** in $CHCl_3$/methanol to slow evaporation in the presence of excess guanidium chloride. Displacement ellipsoids are scaled to the 50% probability level.

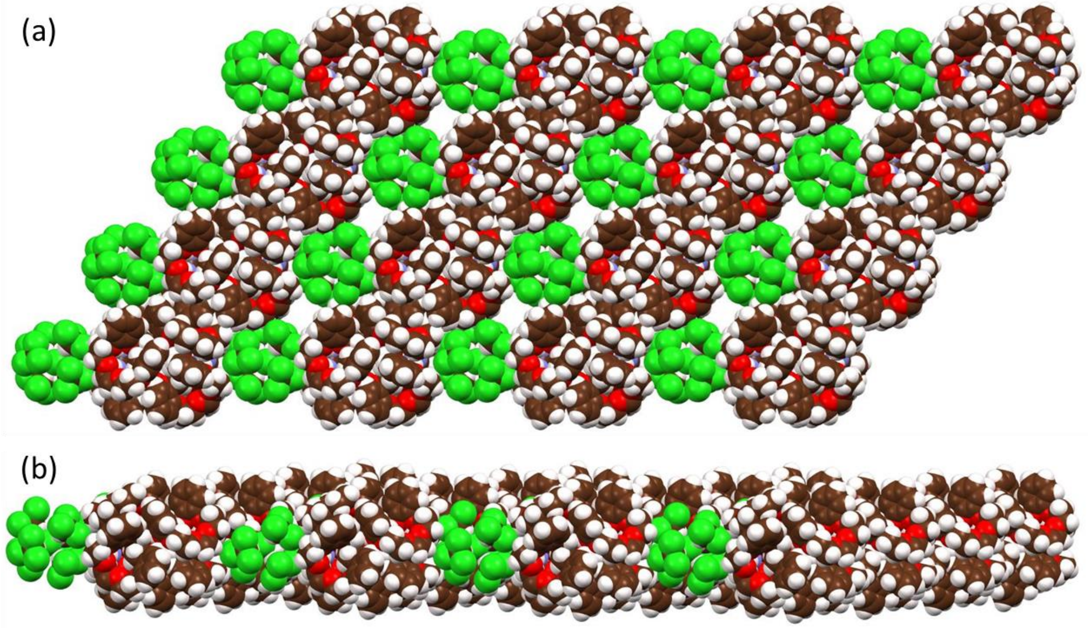

Fig. S9. (a) Top and (b) front views of the highly ordered 2D networks formed in the solid state that comprise subunits of complex $\mathbf{2}_2 \cdot CN_3H_6^+ \cdot CN_3H_5$ complex and $[Cl(CHCl_3)_6]^-$ clusters. All molecules are shown in space-filling form.

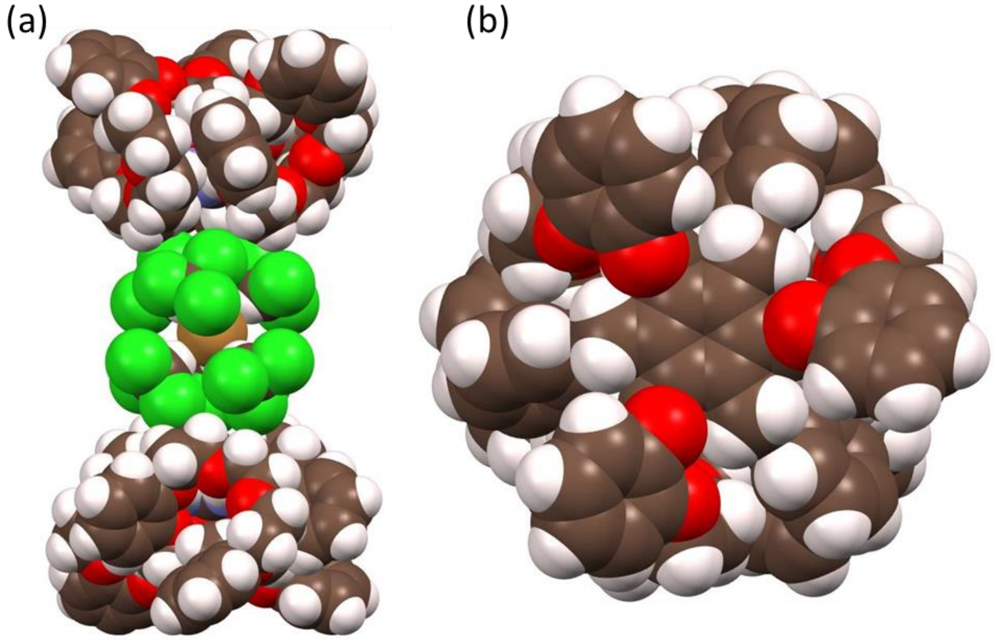

Fig. S10. (a) Front and (b) top views of single-crystal structure of complex **2**$\cdot CN_3H_6^+\cdot CN_3H_5\cdot[Br(CHCl_3)_6]^-$ shown in space-filling form. Single crystals used for this analysis were grown by subjecting a solution of **2** in $CHCl_3$/methanol to slow evaporation in the presence of excess guanidium bromide. Displacement ellipsoids are scaled to the 50% probability level.

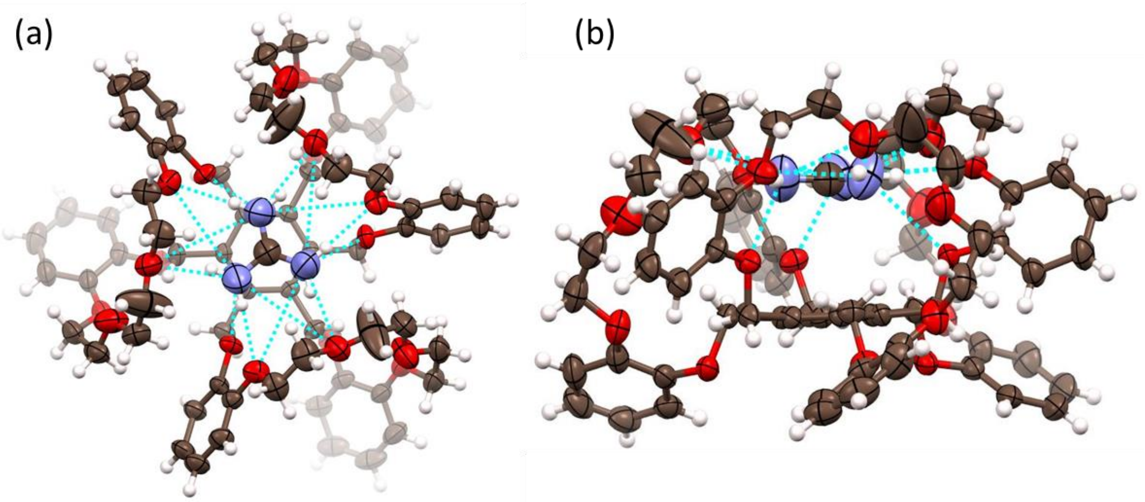

Fig. S11. (a) Top view and (b) front views of crystal structure of complex **2**$\cdot 0.5CN_3H_6^+\cdot 0.5CN_3H_5$ shown in ellipsoid form. Single crystals used for this analysis were grown by subjecting a solution of **2** in $CHCl_3$/methanol to slow evaporation in the presence of excess guanidium bromide. Displacement ellipsoids are scaled to the 50% probability level. Inferred hydrogen bonds are indicated by blue dotted lines.

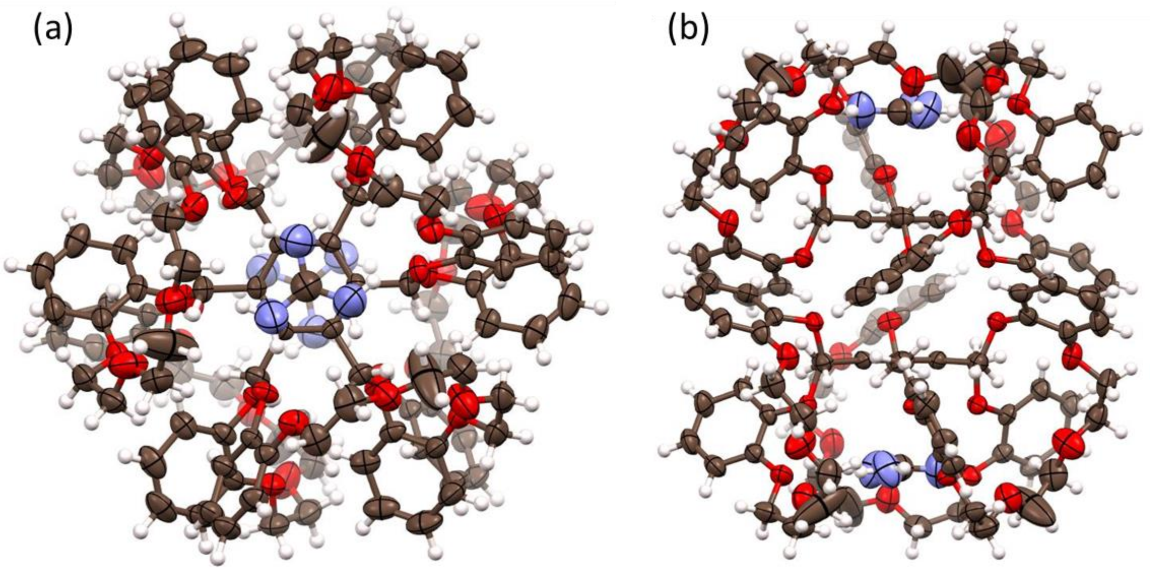

Fig. S12. (a) top view and (b) front view of dimeric complex **2**$_2$·$CN_3H_6^+$·CN_3H_5 shown in ellipsoid form. Single crystals used for this analysis were grown by subjecting a solution of **2** in $CHCl_3$/methanol to slow evaporation in the presence of excess guanidium bromide. Displacement ellipsoids are scaled to the 50% probability level.

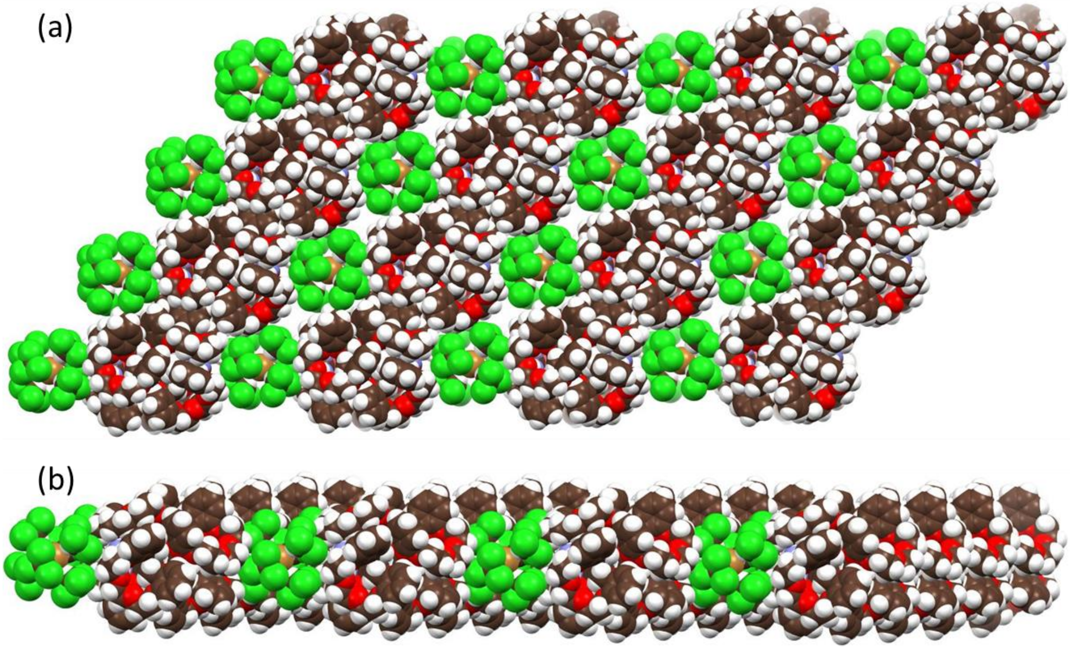

Fig. S13. (a) Top and (b) front views of the highly ordered 2D networks containing complex **2**$_2$·$CN_3H_6^+$·CN_3H_5 and $[Br(CHCl_3)_6]^-$ clusters. All molecules are shown in space-filling form.

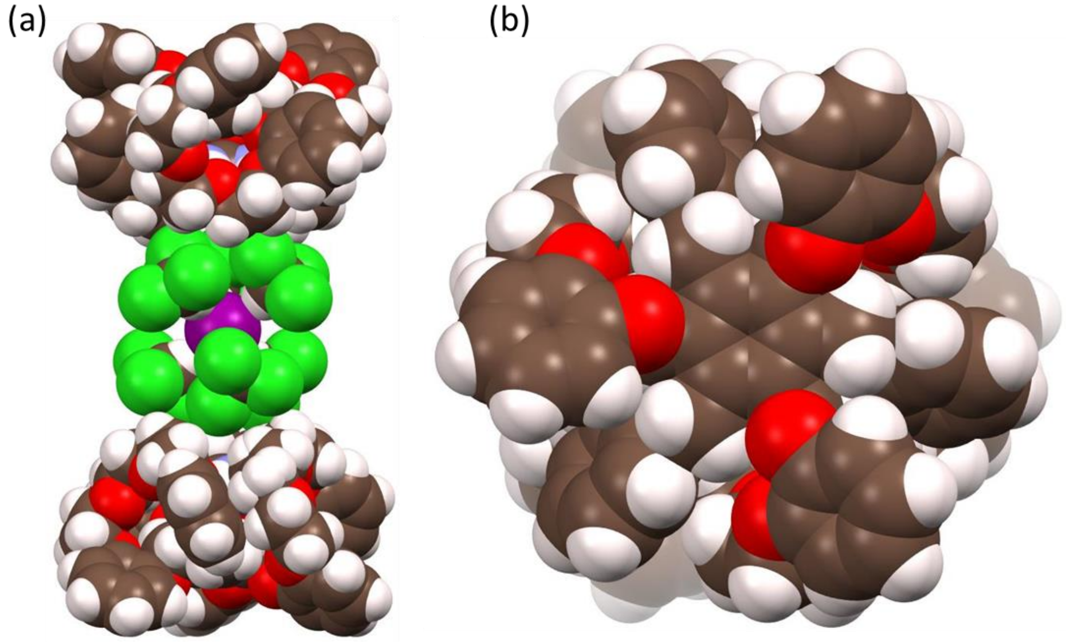

Fig. S14. (a) Front and (b) top views of single-crystal structure of complex **2**$\cdot CN_3H_6^+ \cdot CN_3H_5 \cdot [I(CHCl_3)_6]^-$ shown in space-filling form. Single crystals used for this analysis were grown by subjecting a solution of **2** in $CHCl_3$/methanol to slow evaporation in the presence of excess guanidium iodide. Displacement ellipsoids are scaled to the 50% probability level.

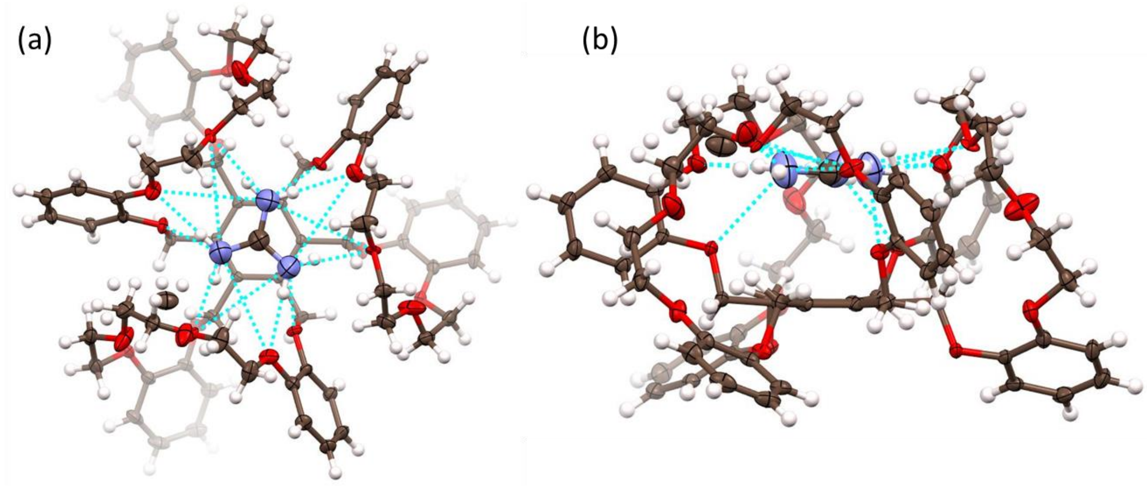

Fig. S15. (a) Top and (b) front views of crystal structure of complex **2**$\cdot 0.5CN_3H_6^+ \cdot 0.5CN_3H_5$ shown in ellipsoid form. Single crystals used for this analysis were grown by subjecting a solution of **2** in $CHCl_3$/methanol to slow evaporation in the presence of excess guanidium iodide. Displacement ellipsoids are scaled to the 50% probability level. Inferred hydrogen bonds are indicated by blue dotted lines.

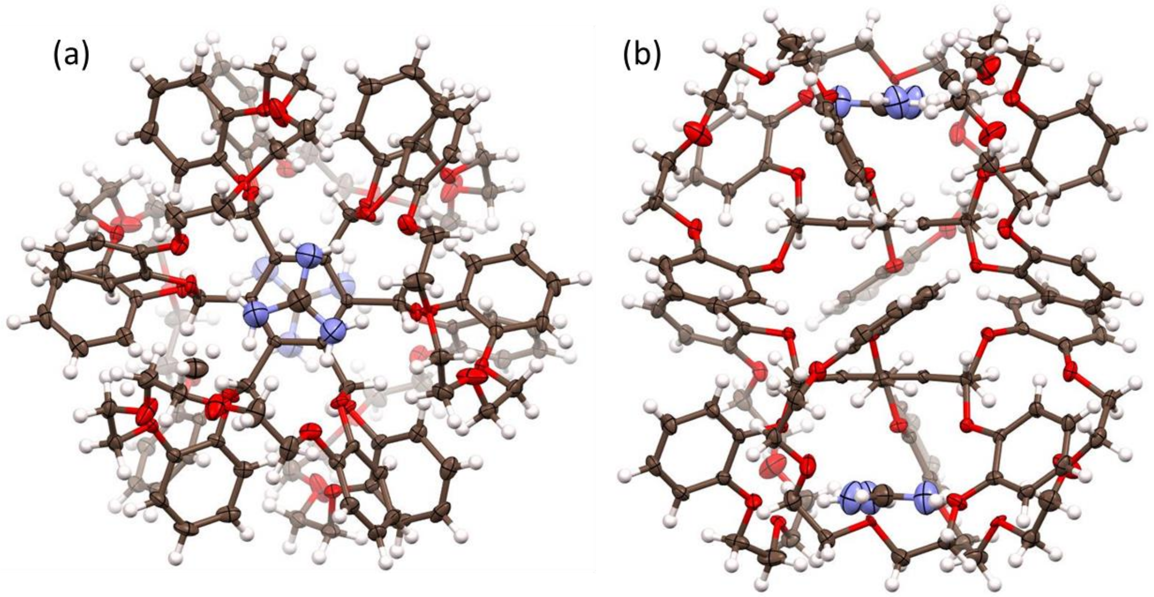

Fig. S16. (a) Top and (b) front views of the dimeric complex $\mathbf{2}_2\cdot CN_3H_6^+\cdot CN_3H_5$ shown in ellipsoid form. Single crystals used for this analysis were grown by subjecting a solution of **2** in $CHCl_3$/methanol to slow evaporation in the presence of excess guanidium iodide. Displacement ellipsoids are scaled to the 50% probability level.

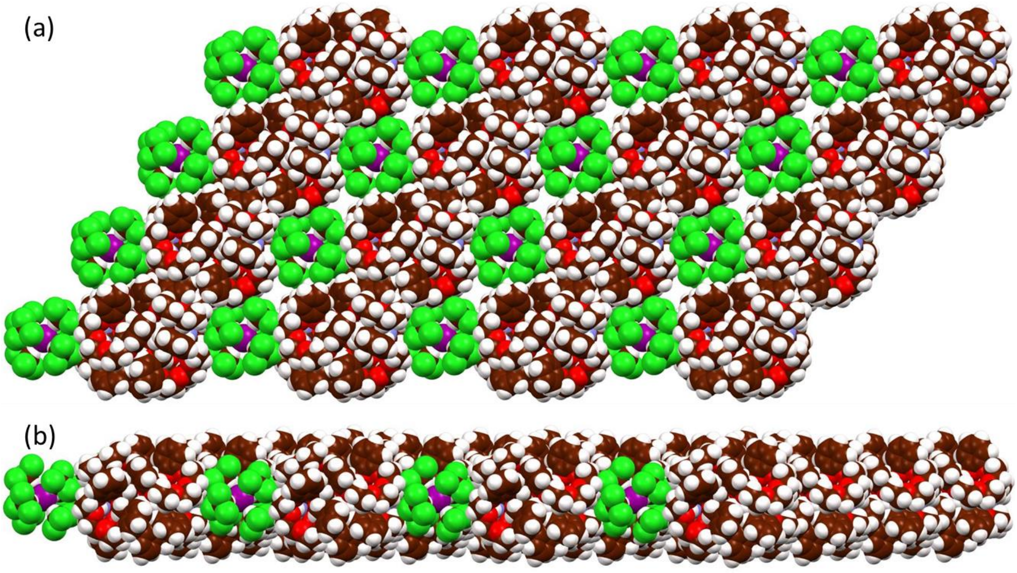

Fig. S17. (a) Top and (b) front views of the highly ordered 2D networks containing complex $\mathbf{2}_2\cdot CN_3H_6^+\cdot CN_3H_5$ and $[I(CHCl_3)_6]^-$ clusters. All molecules are shown in space-filling form.

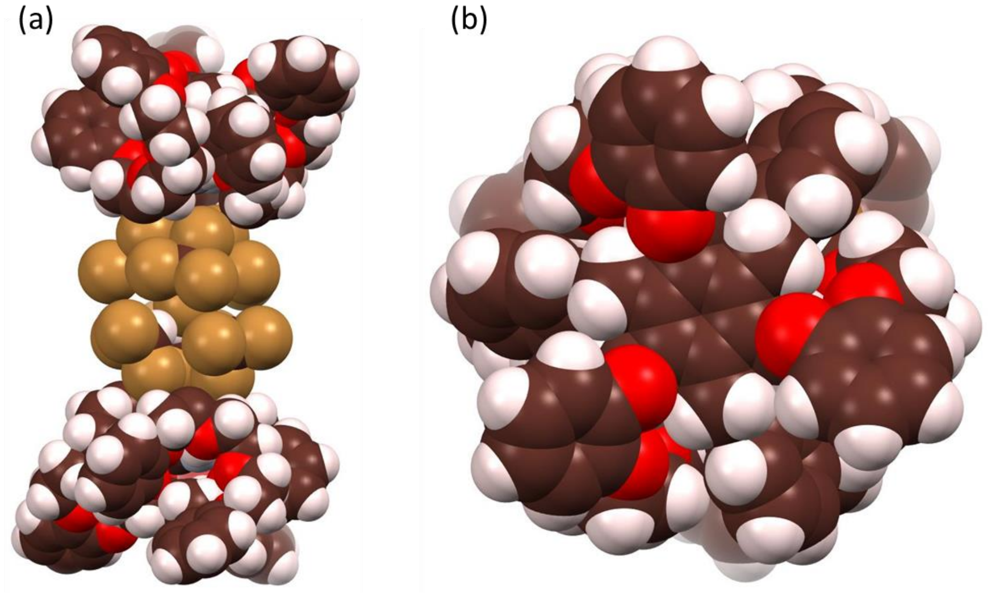

Fig. S18. (a) Front and (b) top views of single-crystal structure of complex **2**$\cdot CN_3H_6^+\cdot CN_3H_5\cdot[Br(CHBr_3)_6]^-$ shown in space-filling form. Single crystals used for this analysis were grown by subjecting a solution of **2** in $CHBr_3$/methanol to slow evaporation in the presence of excess guanidium chloride. Displacement ellipsoids are scaled to the 50% probability level. Although guanidium chloride was used, the counter anions were found to be bromide that might come from the decomposition of $CHBr_3$.

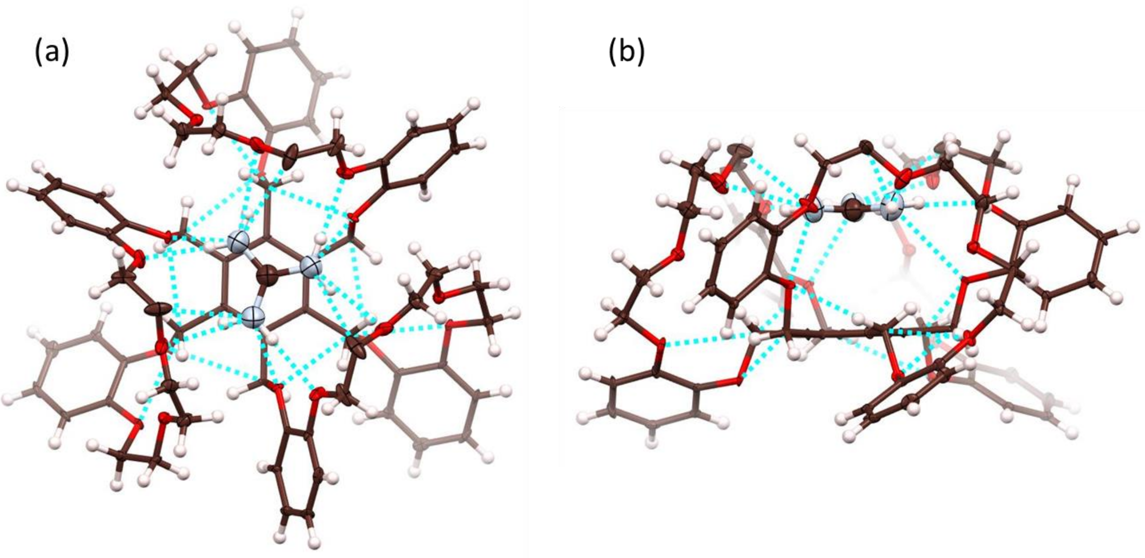

Fig. S19. (a) Top and (b) front views of crystal structure of complex **2**$\cdot 0.5CN_3H_6^+\cdot 0.5CN_3H_5$ shown in ellipsoid form. Single crystals used for this analysis were grown by subjecting a solution of **2** in $CHBr_3$/methanol to slow evaporation in the presence of excess guanidium chloride. Displacement ellipsoids are scaled to the 50% probability level. Inferred hydrogen bonds are indicated by blue dotted lines. Although guanidium chloride was used, the counter anions were found to be bromide that might come from the decomposition of $CHBr_3$.

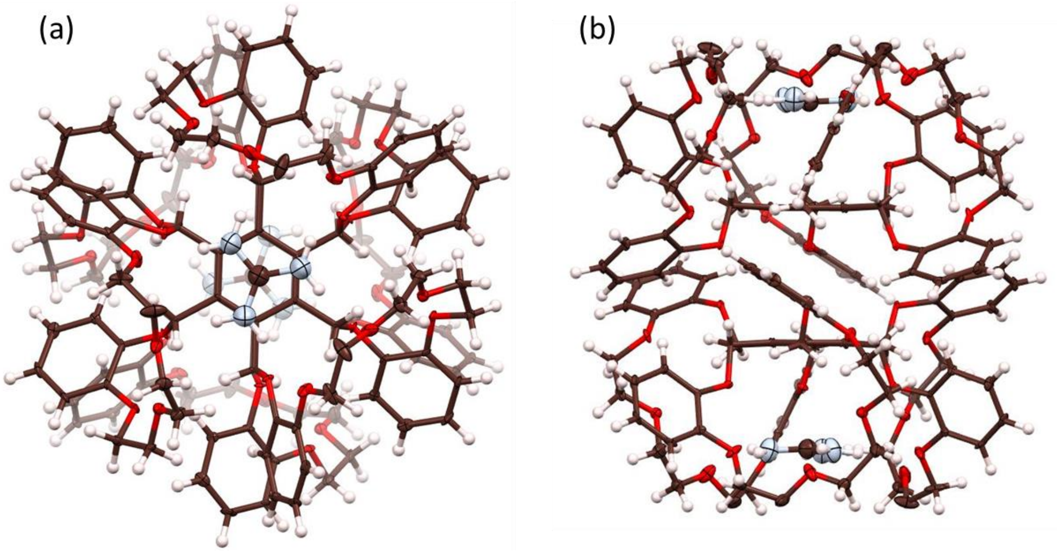

Fig. S20. (a) Top and (b) front views of the dimeric complex $\mathbf{2}_2 \cdot CN_3H_6^+ \cdot CN_3H_5$ shown in ellipsoid form. Single crystals used for this analysis were grown by subjecting a solution of **2** in $CHBr_3$/methanol to slow evaporation in the presence of excess guanidium iodide. Displacement ellipsoids are scaled to the 50% probability level. Although guanidium chloride was used, the counter anions were found to be bromide that might come from the decomposition of $CHBr_3$.

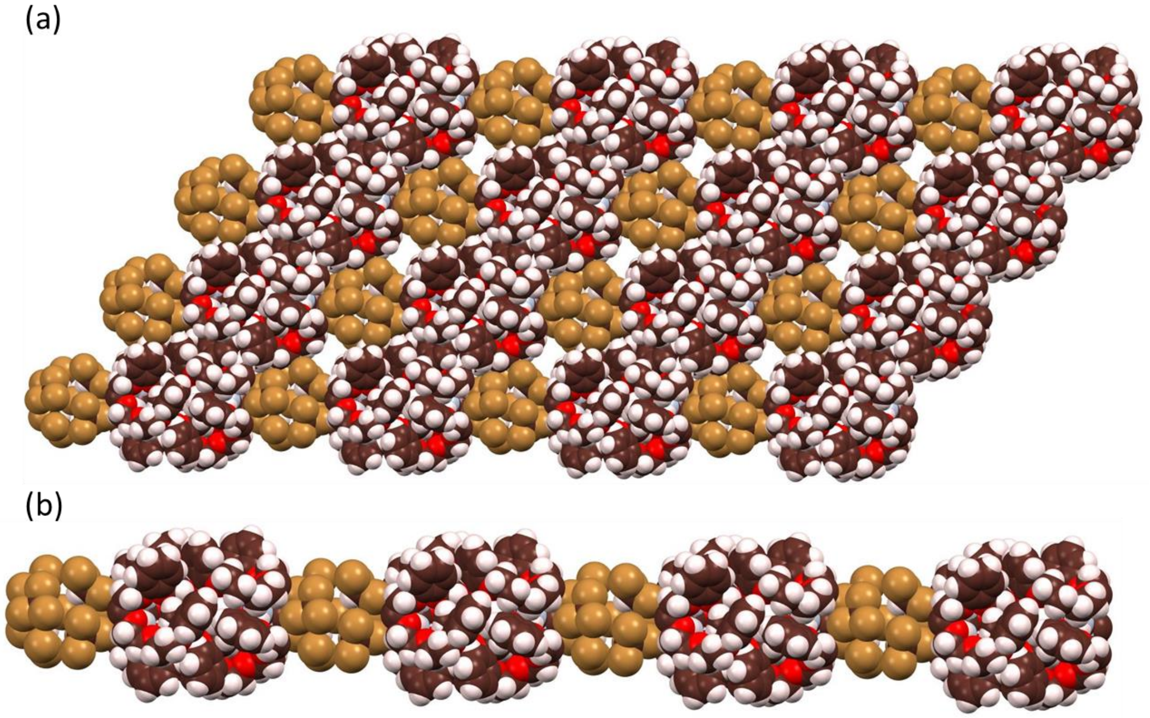

Fig. S21. (a) Top and (b) front views of the highly ordered 2D networks containing complex $\mathbf{2}_2 \cdot CN_3H_6^+ \cdot CN_3H_5$ and $[Br(CHBr_3)_6]^-$ clusters. All molecules are shown in space-filling form.

4. Rapid crystallization experimental

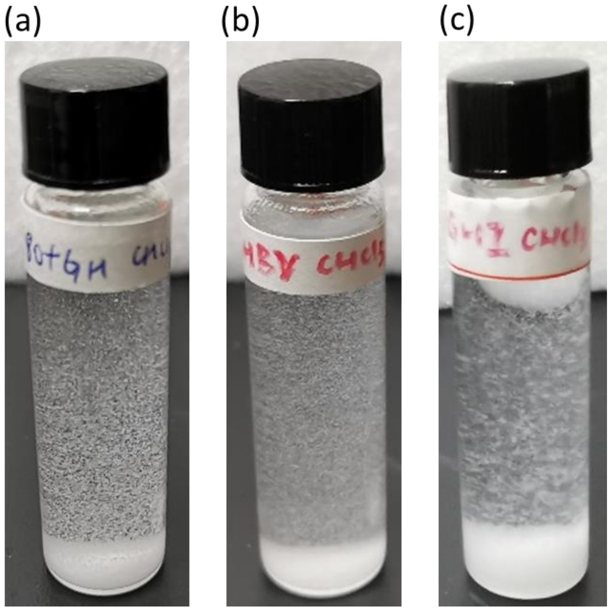

Fig. S22. Photographs of the precipitates formed when hexanes was injected into $CHCl_3/CH_3OH$ (2:1, v/v) solutions of **2** in the presence of (a) guanidium chloride, (b) guanidium bromide, and (c) guanidium iodide in a molar ratio of 1:1.

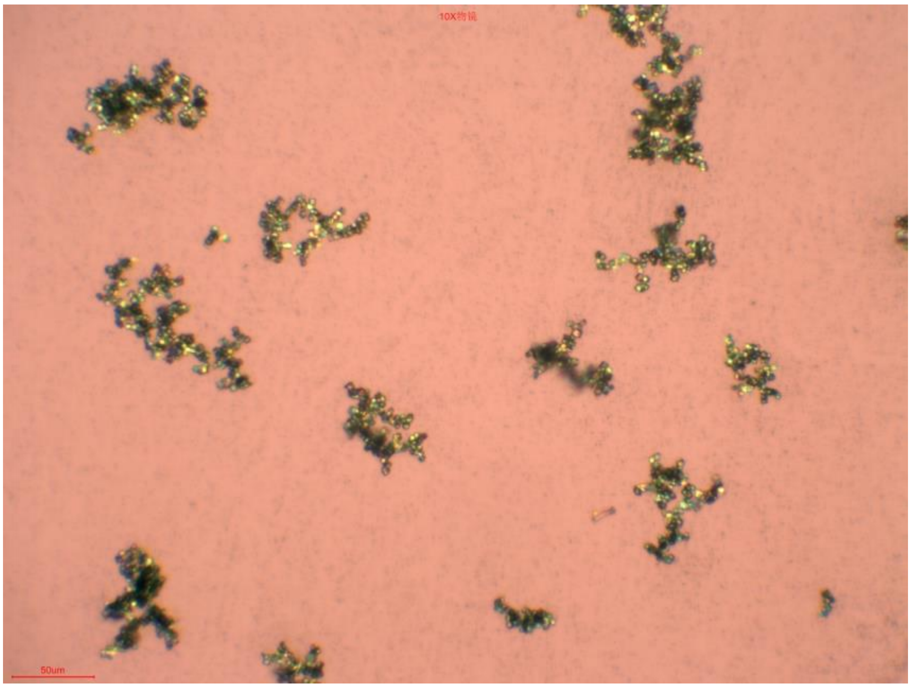

Fig. S23. Polarizing microscope image (crossed polarizers, ×10) of the crystalline entities presumed to be formed upon injection of hexanes into a $CHCl_3/CH_3OH$ (9:1, v/v) solution of **2** in the presence of guanidium chloride in a molar ratio of 1:1 as per Fig. S22a.

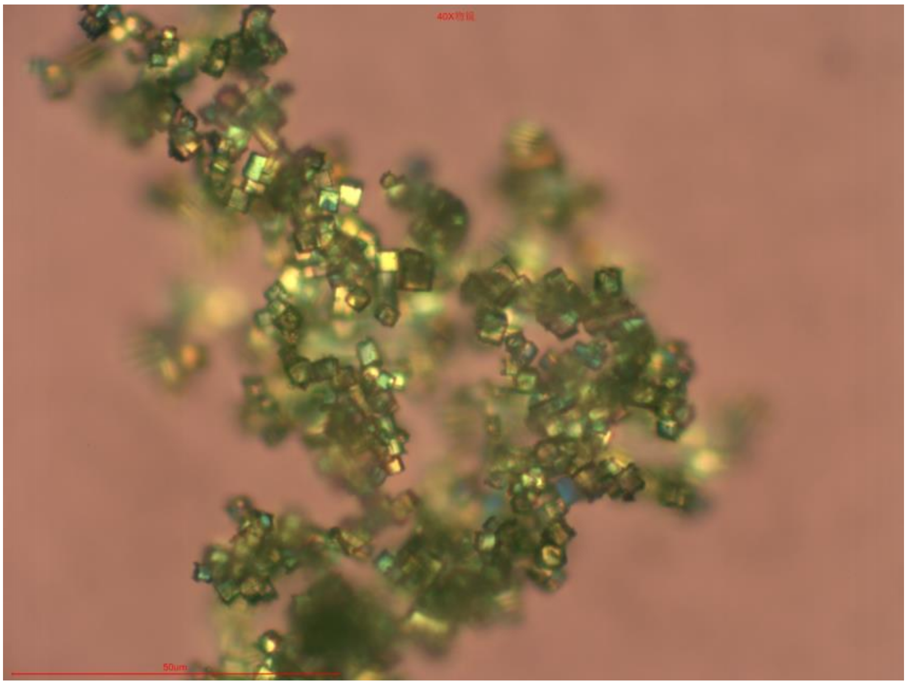

Fig. S24. Polarizing microscope image (crossed polarizers, ×40) of the crystalline entities presumed to be formed upon injection of hexanes into a $CHCl_3/CH_3OH$ (9:1, v/v) solution of **2** in the presence of guanidium chloride in a molar ratio of 1:1 as per Fig. S22a.

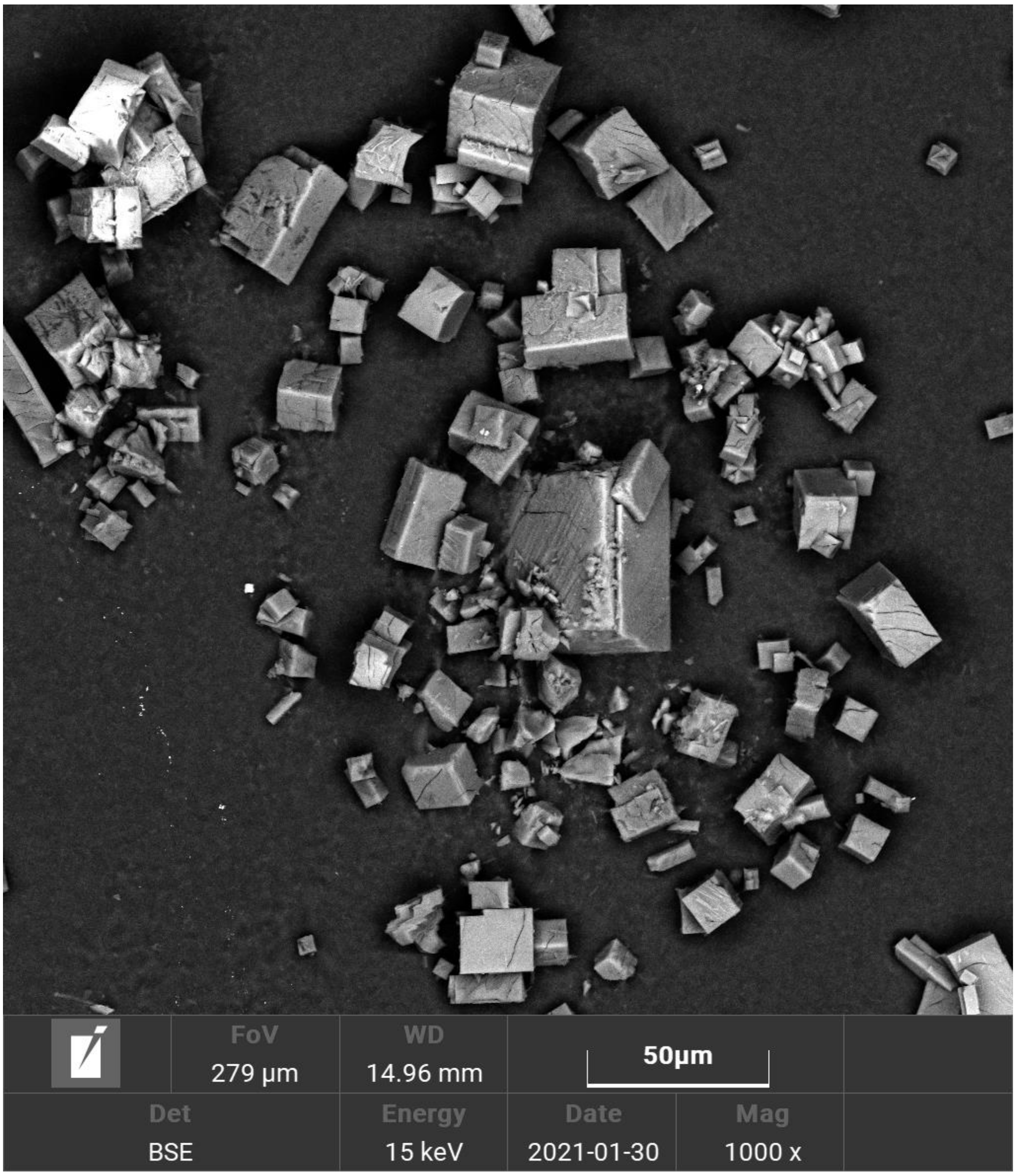

Fig. S25. SEM image of the crystalline entities presumed to be formed upon injection of hexanes into a $CHCl_3/CH_3OH$ (9:1, v/v) solution of **2** in the presence of guanidium chloride in a molar ratio of 1:1 per Fig. S22a without any sputter coating.

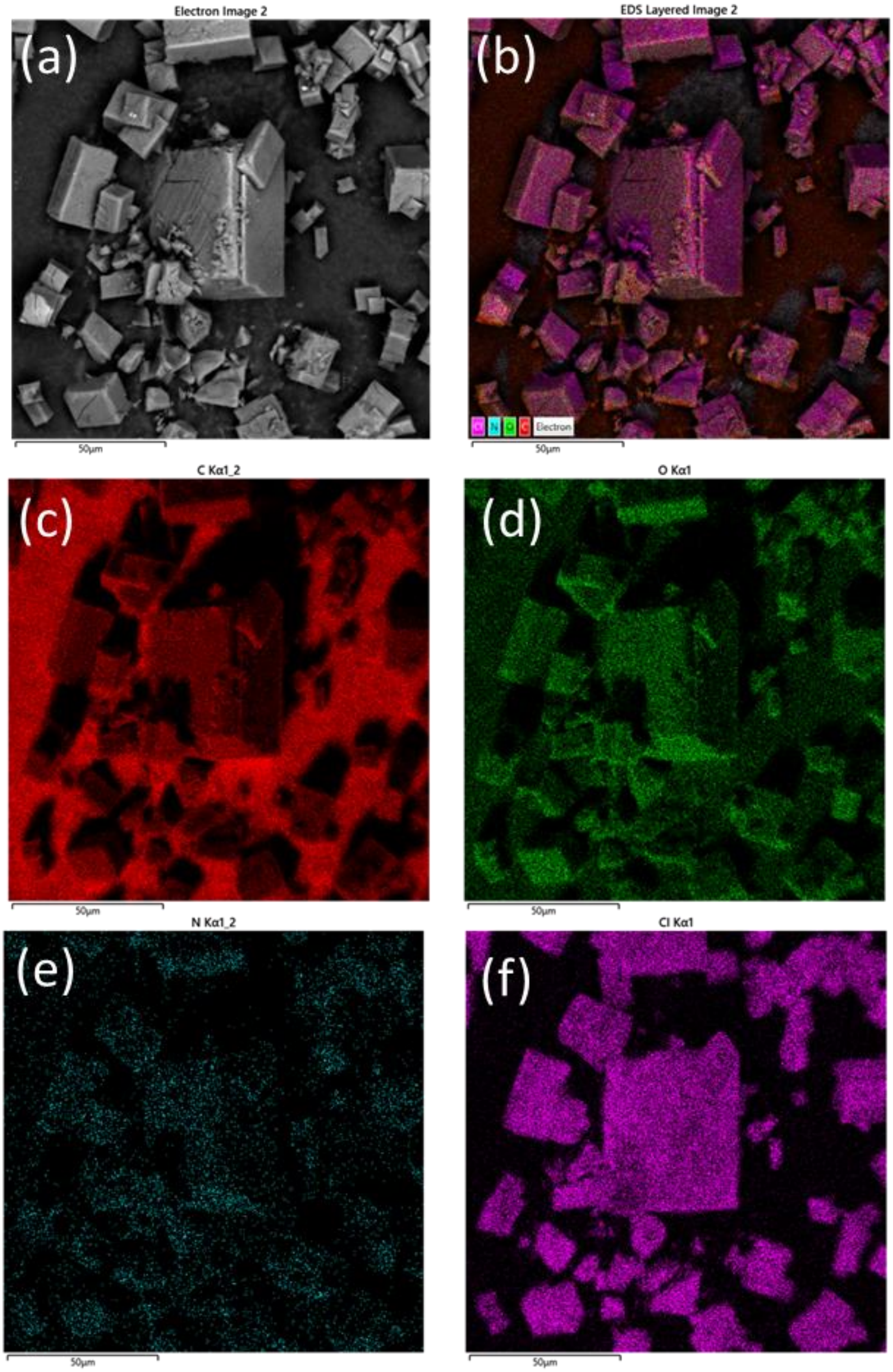

Fig. S26. (a) SEM image of the crystalline entities formed from **2** and guanidium chloride and SEM/EDS mapping for (b) C + N + O + Cl + electron overlay, (c) C, (d) N, (e) O, and (f) Cl.

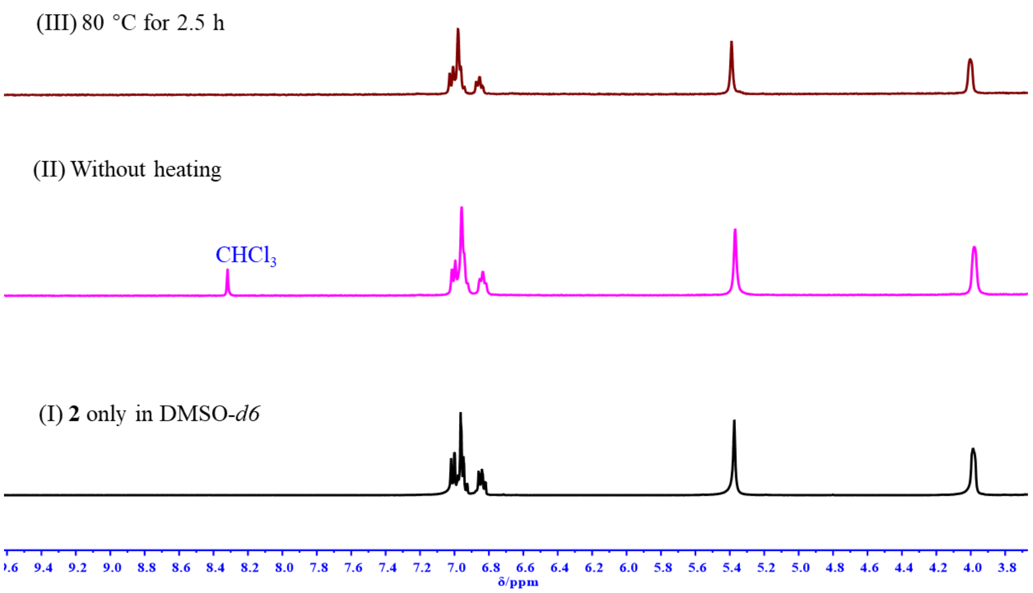

Fig. S27. Selected regions of the ^{1}H NMR spectra of solutions of receptor **2** in DMSO-d_6 (I) and particles obtained by mixing **2** with guanidium chloride in the presence of $CHCl_3$ under conditions of rapid precipitation without heating (II) or treated at 80 °C for 2.5 h (III), with both later solid samples redissolved in DMSO-d_6.

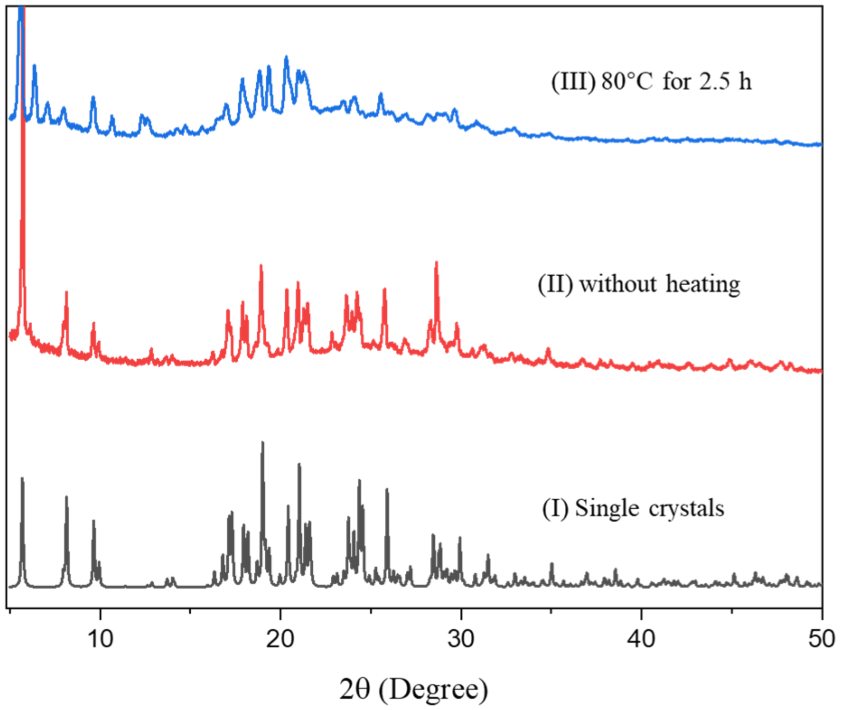

Fig. S28. (I) Simulated PXRD pattern using the data from the single-crystal structure of complex **2**·$CN_3H_6^+$·CN_3H_5·$[Cl(CHCl_3)_6]^-$ and PXRD patterns of the particles obtained by mixing **2** with guanidium chloride in the presence of $CHCl_3$ under conditions of rapid precipitation without heating (II) or after heating at 80 °C for 2.5 h (III).

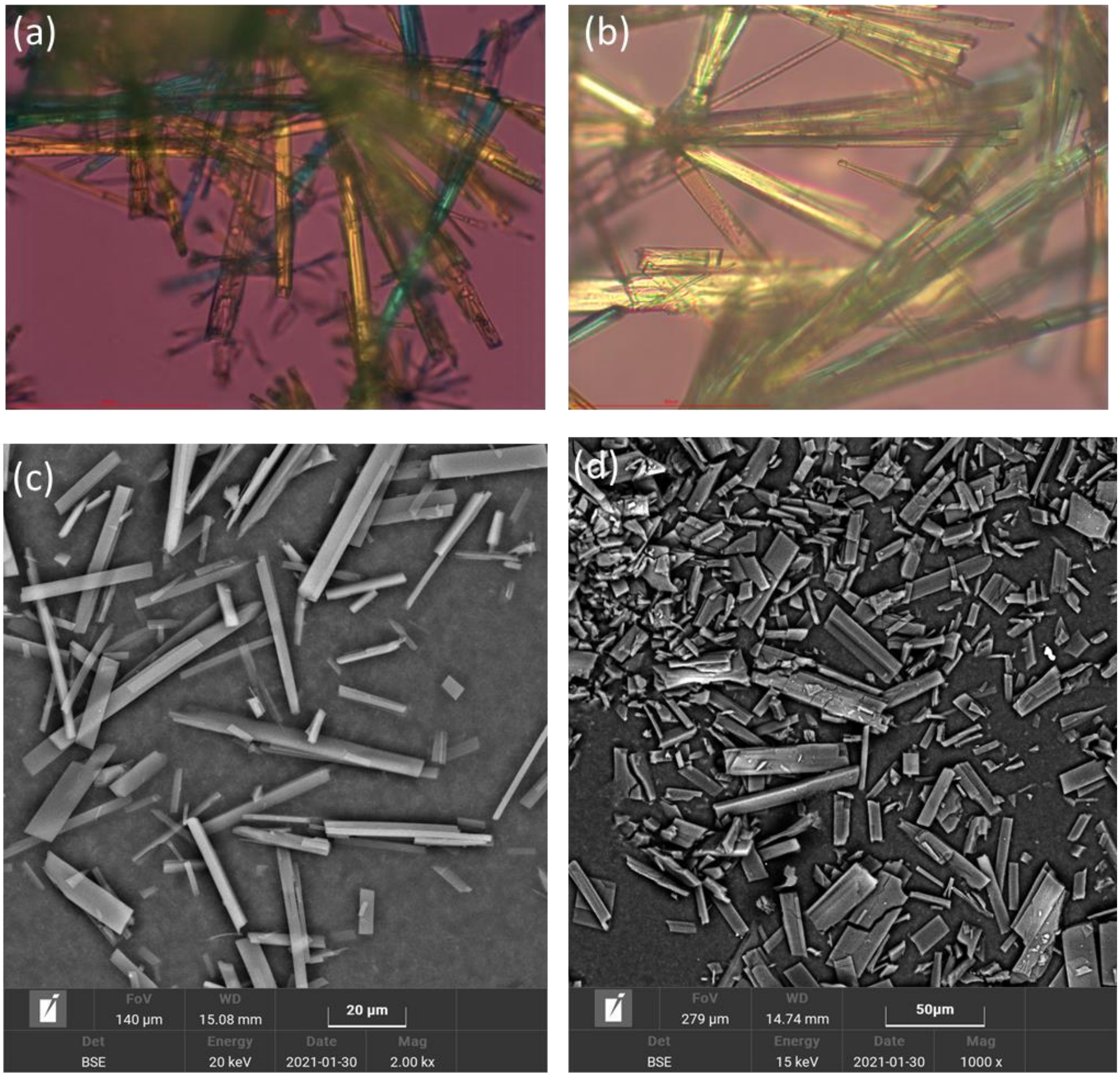

Fig. S29. Polarizing microscope images (crossed polarizers, ×40) of the crystalline entities presumed to be formed upon injection of hexanes into (a) a $CHCl_3/CH_3OH$ (9:1, v/v) solution of **2** and (b) a CH_2Cl_2/CH_3OH (9:1, v/v) solution of **2**. SEM images of the crystalline entities presumed to be formed upon injection of hexanes into (c) a $CHCl_3/CH_3OH$ (9:1, v/v) solution of **2** and (d) a CH_2Cl_2/CH_3OH (9:1, v/v) solution of **2**. In both cases, column-like crystalline entities formed by receptor **2** were observed.

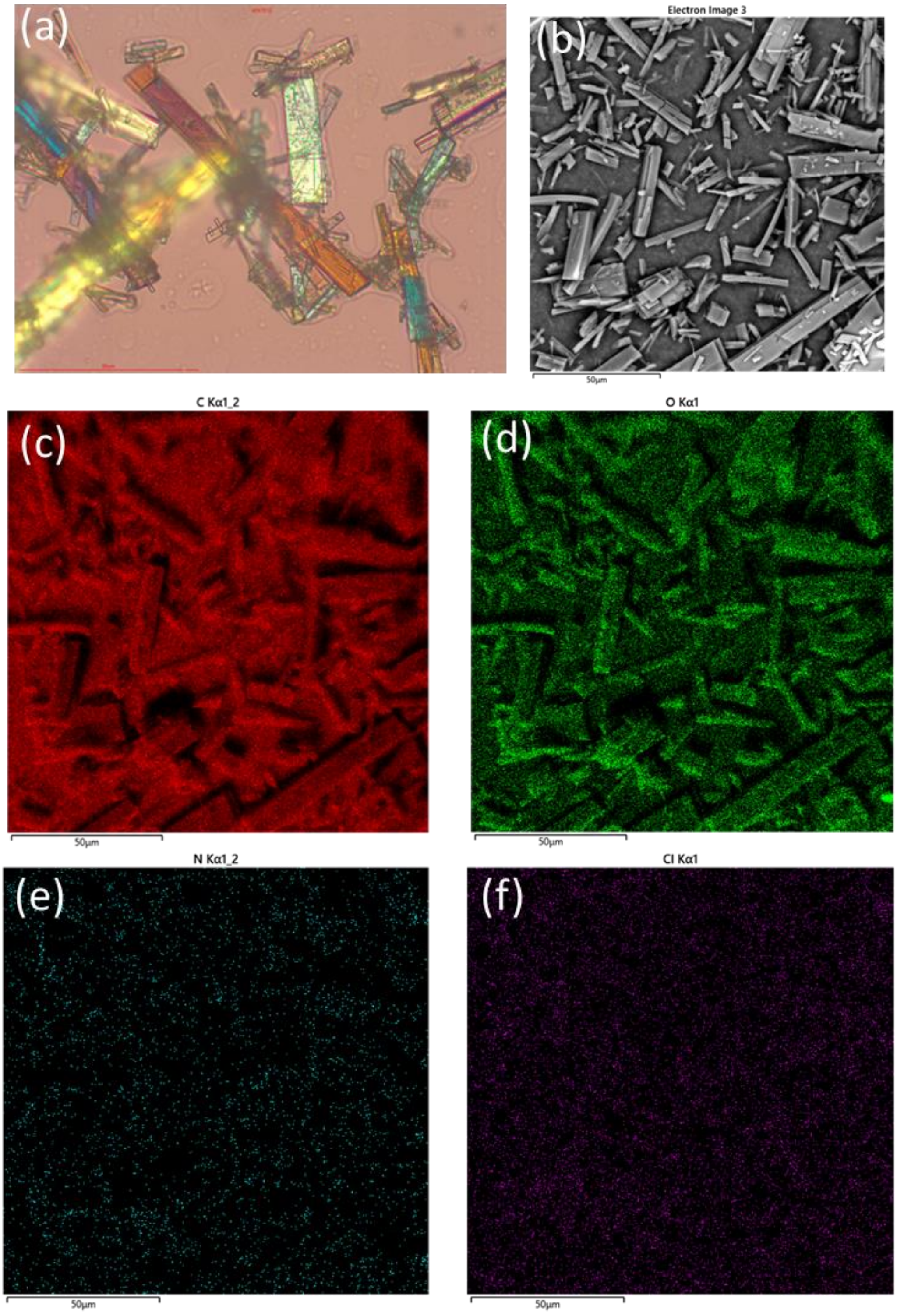

Fig. S30. (a) Polarizing microscope image (crossed polarizers, ×40) and (b) SEM image of the crystalline entities presumed to be formed upon injection of hexanes into a CH_2Cl_2/CH_3OH (9:1, v/v) solution of **2** in the presence of guanidium chloride and SEM/EDS mapping for (c) C, (d) N, (e) O, and (f) Cl. Under these conditions, the microcrystalline material obtained consists of only receptor **2**.

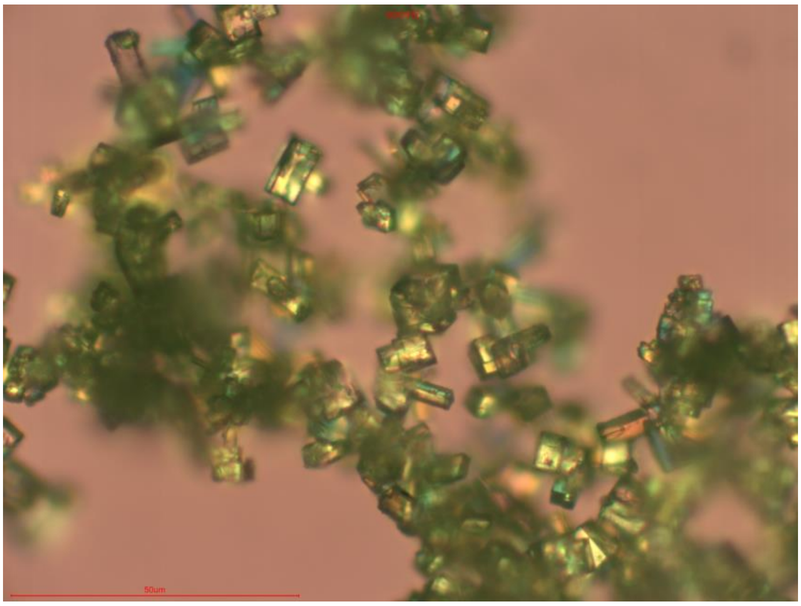

Fig. S31. Polarizing microscope image (crossed polarizers, ×40) of the crystalline entities presumed to be formed upon injection of hexanes into a $CHCl_3/CH_3OH$ (9:1, v/v) solution of **2** in the presence of guanidium bromide in a molar ratio of 1:1 produced *in situ* per Fig. S22b.

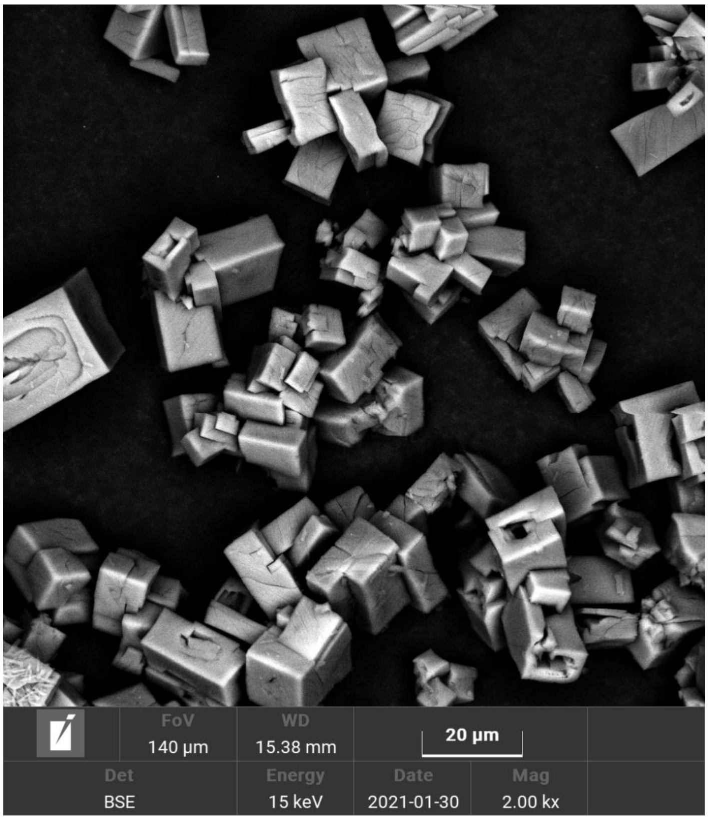

Fig. S32. SEM image of the crystalline entities presumed to be formed upon injection of hexanes into a $CHCl_3/CH_3OH$ (9:1, v/v) solution of **2** in the presence of guanidium bromide in a molar ratio of 1:1 produced *in situ* per Fig. S22b without any sputter coating.

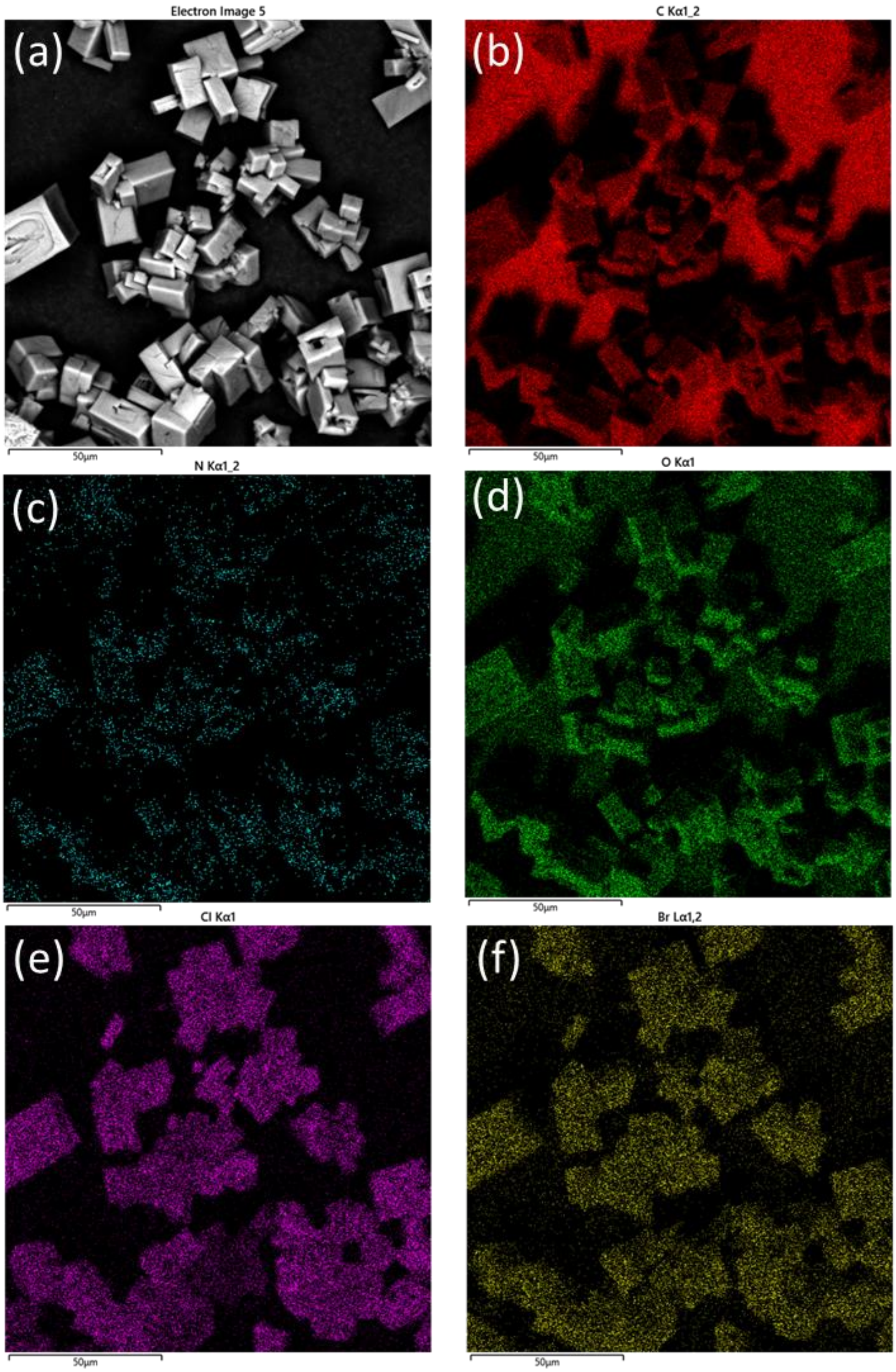

Fig. S33. (a) SEM image of the crystalline entities formed from **2** and guanidium bromide and SEM/EDS mapping for (b) C, (c) N, (d) O, (e) Cl, and (f) Br. These observations provide support for the conclusion that the crystalline ensembles produced under these conditions contain not only receptor **2** and guanidium bromide, but also $CHCl_3$.

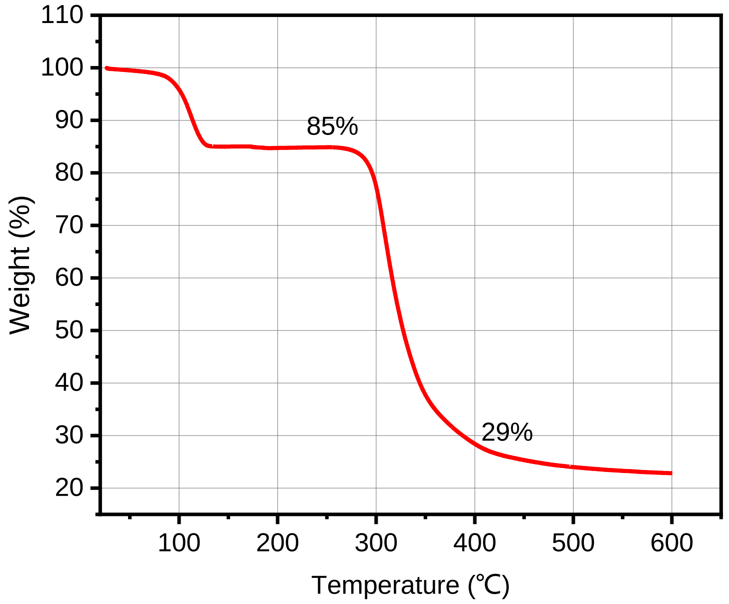

Fig. S34. Thermogravimetric decomposition trace for the crystalline particles presumed to be formed upon injection of hexanes into a $CHCl_3/CH_3OH$ (9:1, v/v) solution of **2** and 1 molar equiv of guanidium bromide produced *in situ* as per Fig. S22b.

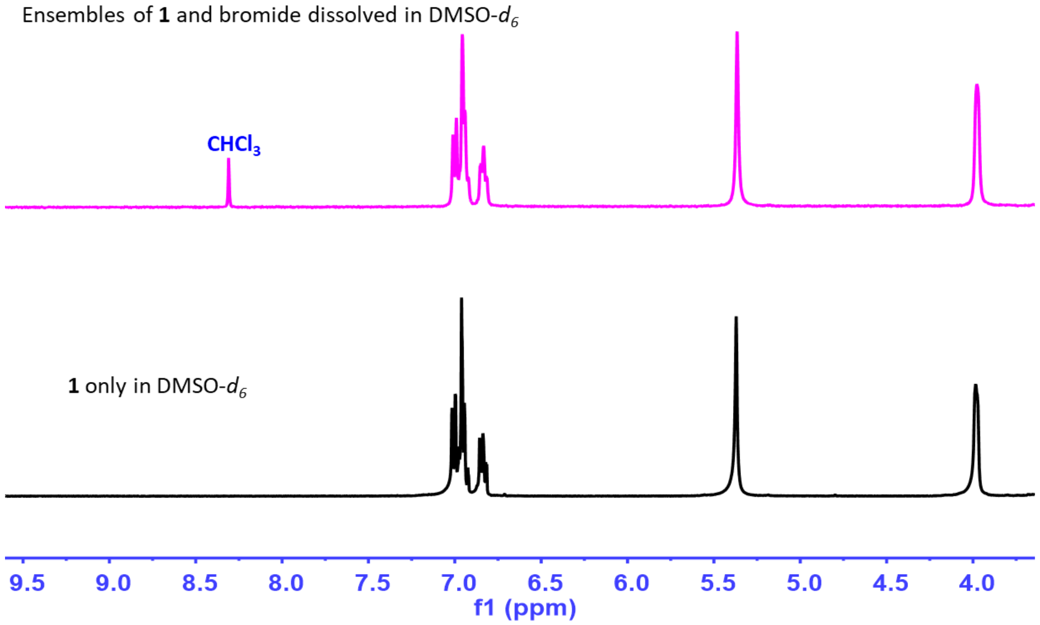

Fig. S35. Selected regions of ^{1}H NMR spectra of solutions of **2** in DMSO-d_6 (bottom) and crystalline particles obtained from Fig. S22b redissolved in DMSO-d_6 (top). A sharp peak corresponding to $CHCl_3$ was observed.

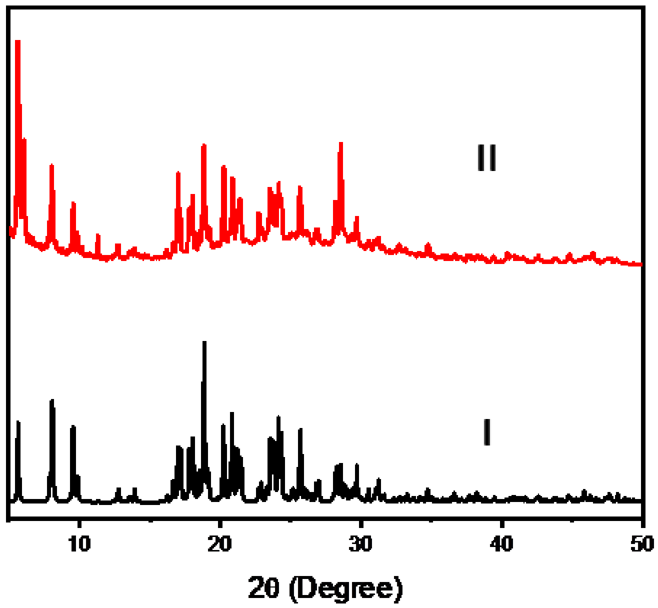

Fig. S36. PXRD patterns of (I) simulated PXRD pattern derived from the single-crystal structural data for the complex **2**$\cdot CN_3H_6^+ \cdot CN_3H_5 \cdot [Cl(CHCl_3)_6]^-$ and (II) crystalline particles presumed to be formed upon injection of hexanes into a $CHCl_3/CH_3OH$ (:/1, v/v) solution of **2** in the presence of guanidium bromide in a 1:1 molar ratio produced *in situ* as per Fig. S22b.

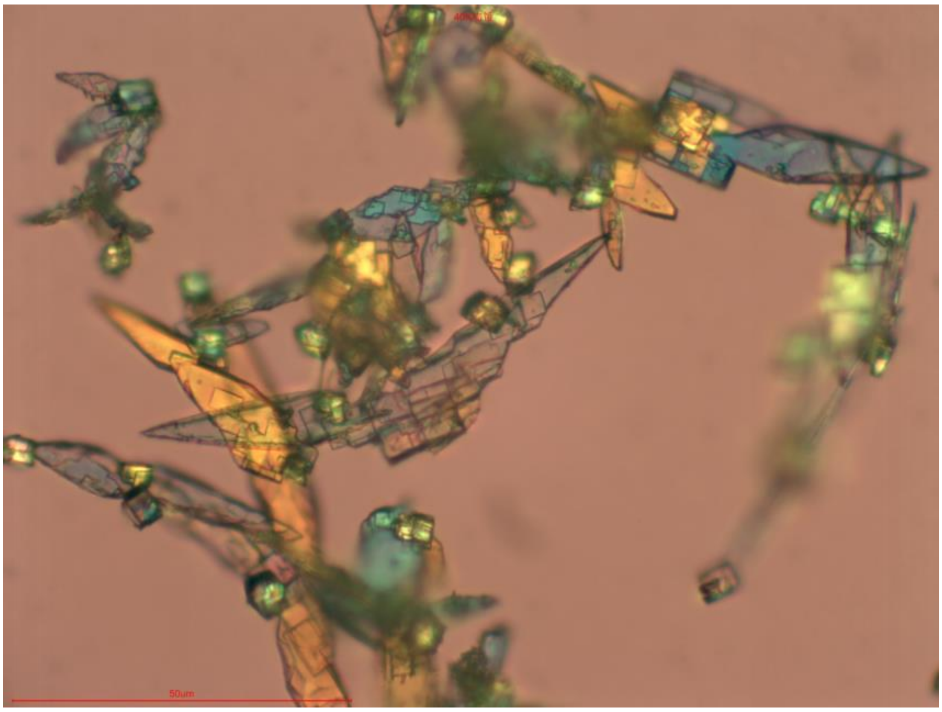

Fig. S37. Polarizing microscope image (crossed polarizers, ×40) of the crystalline entities presumed to be formed upon injection of hexanes into a $CHCl_3/CH_3OH$ (9:1, v/v) solution of **2** in the presence of guanidium iodide in a 1:1 molar ratio produced *in situ* as per Fig. S22c.

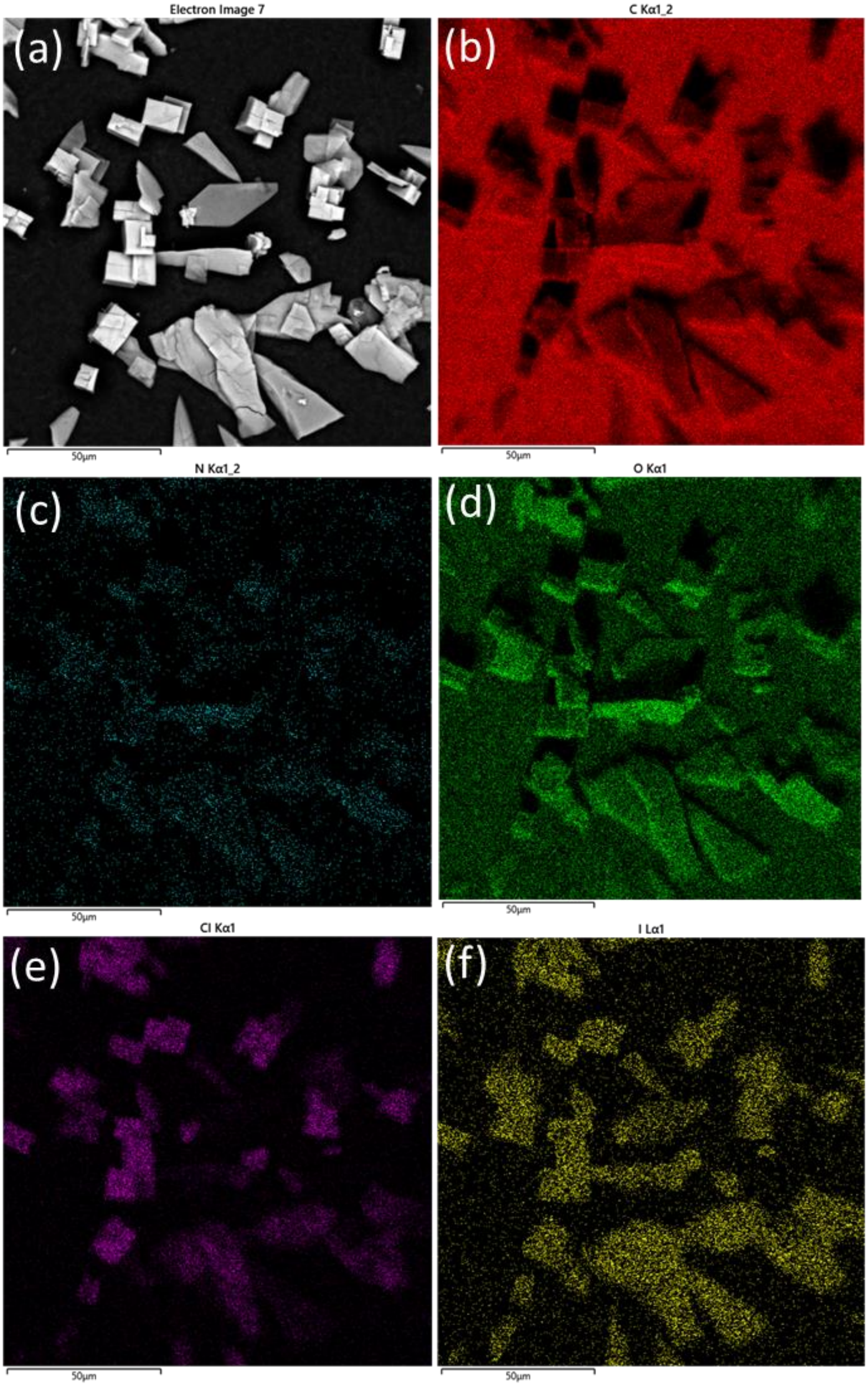

Fig. S38. (a) SEM image of the crystalline entities presumed to be formed from **2** and guanidium iodide and SEM/EDS mapping for (b) C, (c) N, (d) O, (e) Cl, and (f) I. These observations provide support for the conclusion that the crystalline ensembles produced under these conditions contain not only receptor **2** and guanidium iodide, but also $CHCl_3$.

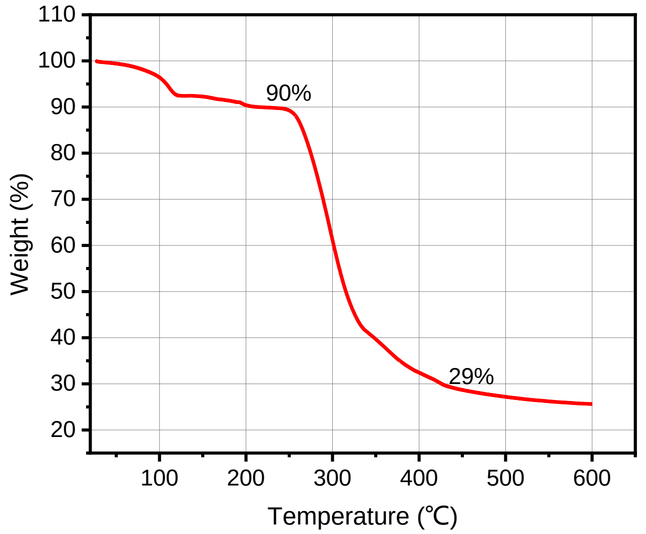

Fig. S39. Thermogravimetric decomposition trace for the crystalline particles presumed to be formed upon injection of hexanes into a $CHCl_3/CH_3OH$ (9:1, v/v) solution of **2** in the presence of guanidium iodide in a 1:1 molar ratio produced *in situ* as per Fig. S22c.

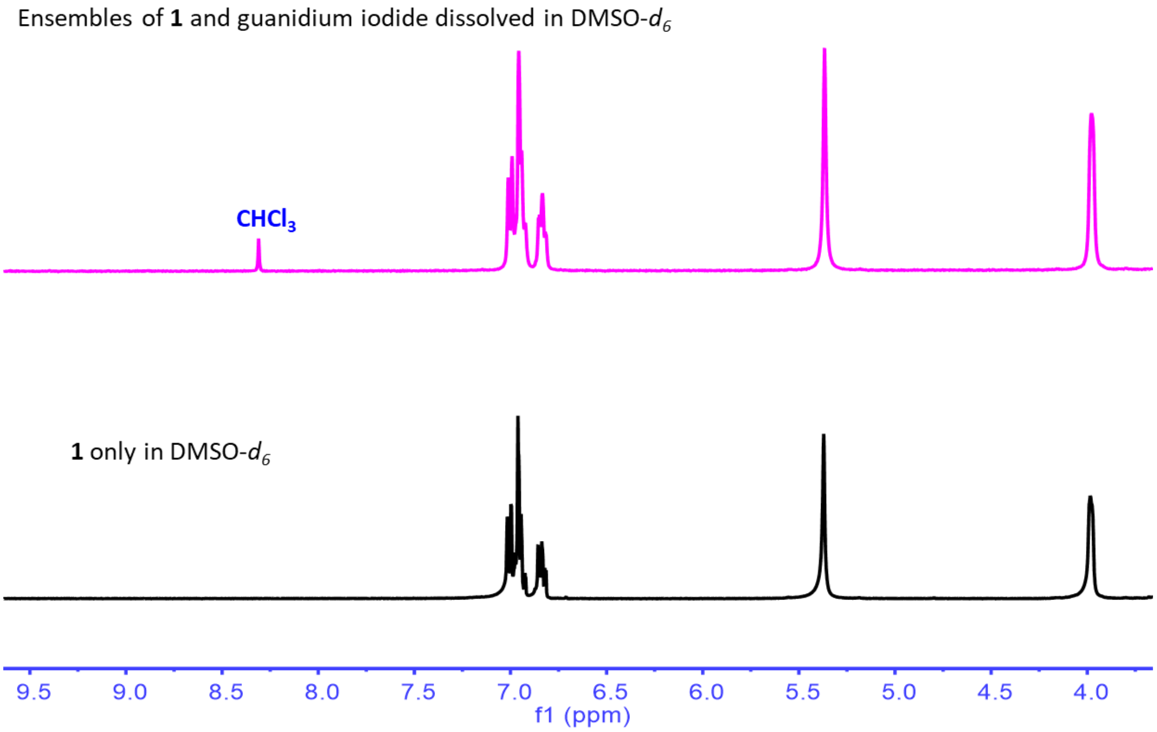

Fig. S40. Selected regions of ^{1}H NMR spectra of solutions of **2** in DMSO-d_6 (bottom) and crystalline particles obtained as described in Fig. S22c and then redissolved in DMSO-d_6 (top). A sharp peak corresponding to $CHCl_3$ was observed.

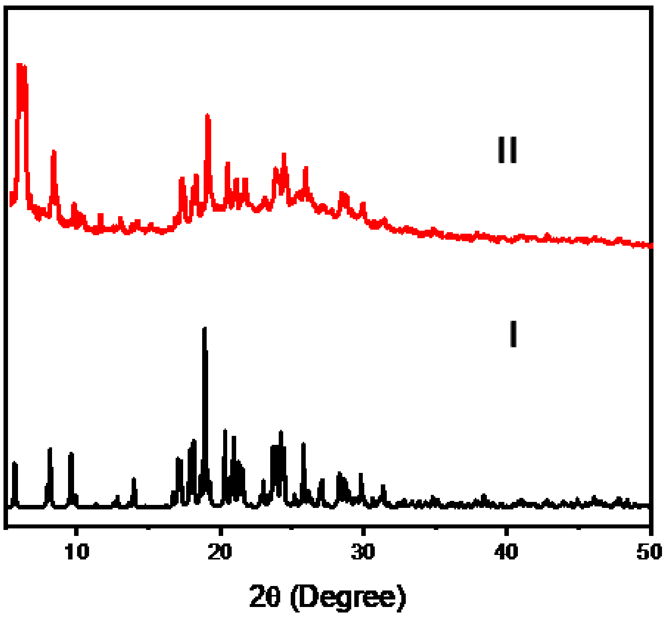

Fig. S41. PXRD patterns of (I) simulated PXRD pattern from single-crystal structure of complex **2**·$CN_3H_6^+$·CN_3H_5·$[Cl(CHCl_3)_6]^-$ and (II) crystalline particles presumed to be formed upon injection of hexanes into a $CHCl_3/CH_3OH$ (9:1, v/v) solution of **2** in the presence of guanidium iodide in a molar ratio of 1:1 produced *in situ* as per Fig. S22c.

5. X-ray experimental details

X-ray experimental for receptor 2

Single crystals of receptor **2** were obtained as colorless plates via the slow evaporation of a $CHCl_3$/CH_3OH solution of receptor **2**. A suitable crystal was selected and the data were collected on a Brucker D8 Advance diffractometer using a µ–focused Mo Kα radiation source (λ = 0.71073 Å). The crystal was kept at 150 K during data collection. Using Olex2,[10] the structure was solved with the ShelXT[11] structure solution program using Direct Methods and refined with the ShelXL[12] refinement package using Least Squares minimization. Tables of positional and thermal parameters, bond lengths and angles, torsion angles are in the CIF file. CCDC deposition number: 2068692.

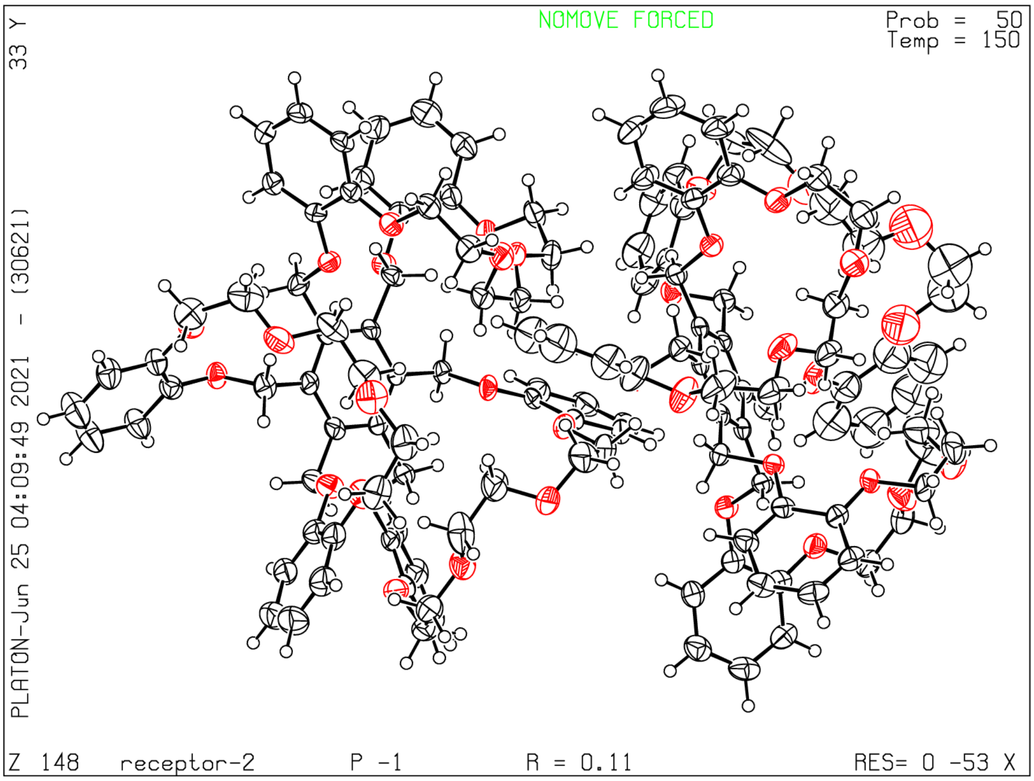

Fig. S42. View of receptor **2**. Displacement ellipsoids are scaled to the 50% probability level

Table S1 Crystal data and structure refinement for receptor-2.

Identification code	receptor-2
Empirical formula	$C_{132}H_{144}O_{36}$
Formula weight	2306.46
Temperature/K	150.15
Crystal system	triclinic
Space group	P-1
a/Å	16.250(8)
b/Å	18.296(7)
c/Å	25.707(12)
α/°	98.113(10)
β/°	91.340(9)
γ/°	108.395(10)
Volume/Å^3	7161(5)
Z	2
ρ_{calc}g/cm^3	1.070
μ/mm^{-1}	0.078
F(000)	2448.0
Crystal size/mm^3	0.438 × 0.188 × 0.139
Radiation	MoKα (λ = 0.71073 Å)
2Θ range for data collection/°	4.282 to 56.74
Index ranges	$-21 \leq h \leq 21$, $-24 \leq k \leq 24$, $-33 \leq l \leq 34$
Reflections collected	78130
Independent reflections	35530 [R_{int} = 0.0949, R_{sigma} = 0.1453]
Data/restraints/parameters	35530/159/1513
Goodness-of-fit on F^2	1.081
Final R indexes [I>=2σ (I)]	R_1 = 0.1093, wR_2 = 0.3100
Final R indexes [all data]	R_1 = 0.1685, wR_2 = 0.3558
Largest diff. peak/hole / e Å^{-3}	1.40/-0.67
CCDC number	2068692

X-ray experimental for complex 2-guanidium chloride

Single crystals of guanidium chloride complex of **2** were obtained as colorless cubes via the slow evaporation of a $CHCl_3/CH_3OH$ solution of receptor **2** in the presence of excess guanidium chloride. A suitable crystal was selected and the data were collected on a Brucker D8 Advance diffractometer using a μ–focused Mo Kα radiation source (λ = 0.71073 Å). The crystal was kept at 150 K during data collection. Using Olex2,[10] the structure was solved with the ShelXT[11] structure solution program using Direct Methods and refined with the ShelXL[12] refinement package using Least Squares minimization. Tables of positional and thermal parameters, bond lengths and angles, torsion angles and figures are in the CIF file. CCDC deposition number: 2068693.

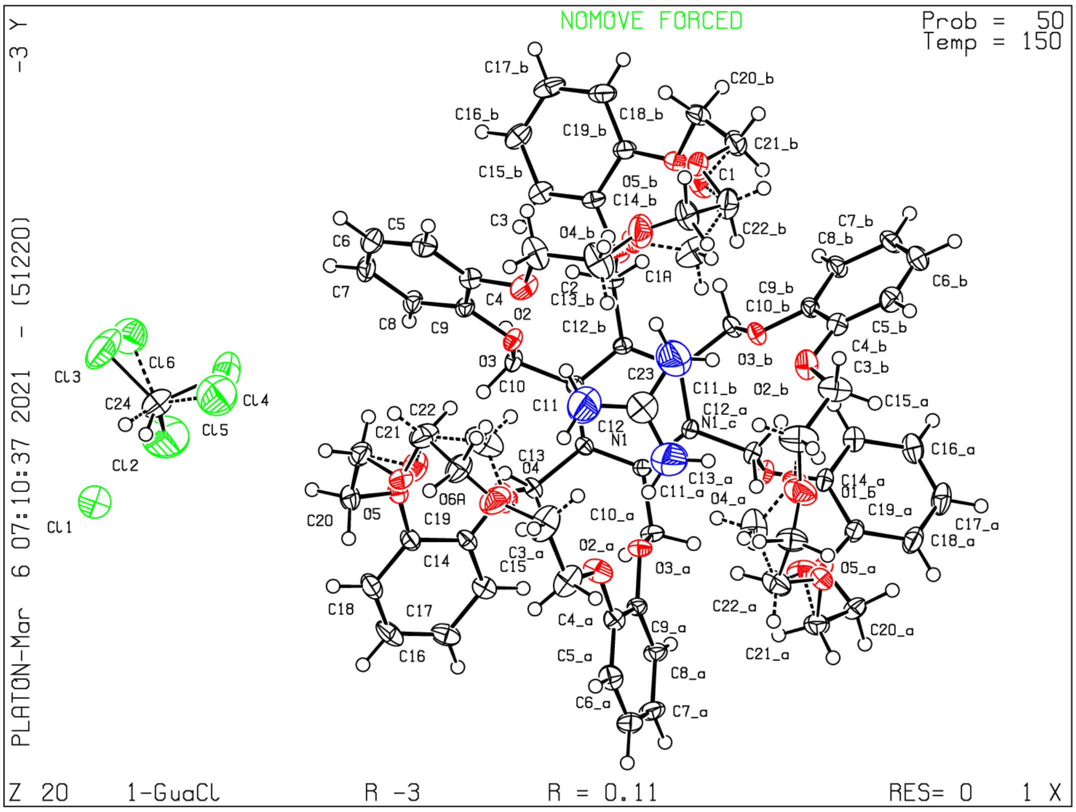

Fig. S43. View of complex $\mathbf{2}\cdot CN_3H_6^+\cdot CN_3H_5\cdot[Cl(CHCl_3)_6]^-$. Displacement ellipsoids are scaled to the 50% probability level.

Table S2 Crystal data and structure refinement for 2-guanidium chloride.

Identification code	2-guanidium chloride
Empirical formula	$C_{140}H_{158}Cl_{19}N_6O_{36}$
Formula weight	3174.22
Temperature/K	150.15
Crystal system	trigonal
Space group	R-3
a/Å	21.694(3)
b/Å	21.694(3)
c/Å	27.480(4)
α/°	90
β/°	90
γ/°	120
Volume/Å^3	11200(3)
Z	3
ρ_{calc}g/cm^3	1.412
μ/mm^{-1}	0.425
F(000)	4953.0
Crystal size/mm^3	0.51 × 0.359 × 0.311
Radiation	MoKα (λ = 0.71073 Å)
2Θ range for data collection/°	4.582 to 60.116
Index ranges	$-23 \leq h \leq 30$, $-30 \leq k \leq 23$, $-38 \leq l \leq 26$
Reflections collected	24200
Independent reflections	7306 [R_{int} = 0.0351, R_{sigma} = 0.0287]
Data/restraints/parameters	7306/24/361
Goodness-of-fit on F^2	1.155
Final R indexes [I>=2σ (I)]	R_1 = 0.1054, wR_2 = 0.2633
Final R indexes [all data]	R_1 = 0.1128, wR_2 = 0.2670
Largest diff. peak/hole / e Å^{-3}	0.53/-0.99
CCDC number	2068693

X-ray experimental for the complex 2-guanidium bromide

Single crystals of guanidium bromide complex of **2** were obtained as colorless cubes via the slow evaporation of a $CHCl_3/CH_3OH$ solution of receptor **2** in the presence of excess guanidium bromide. A suitable crystal was selected and the data were collected on an Agilent Technologies SuperNova Dual Source diffractometer using a μ-focus CuKα radiation source (λ = 1.5418 Å) with collimating mirror monochromators. The crystal was kept at 293.15 K during data collection. The crystal was kept at 150 K during data collection. Using Olex2,[10] the structure was solved with the ShelXT[11] structure solution program using Direct Methods and refined with the ShelXL[12] refinement package using Least Squares minimization. Tables of positional and thermal parameters, bond lengths and angles, torsion angles and figures are in the CIF file. CCDC deposition number: 2068694.

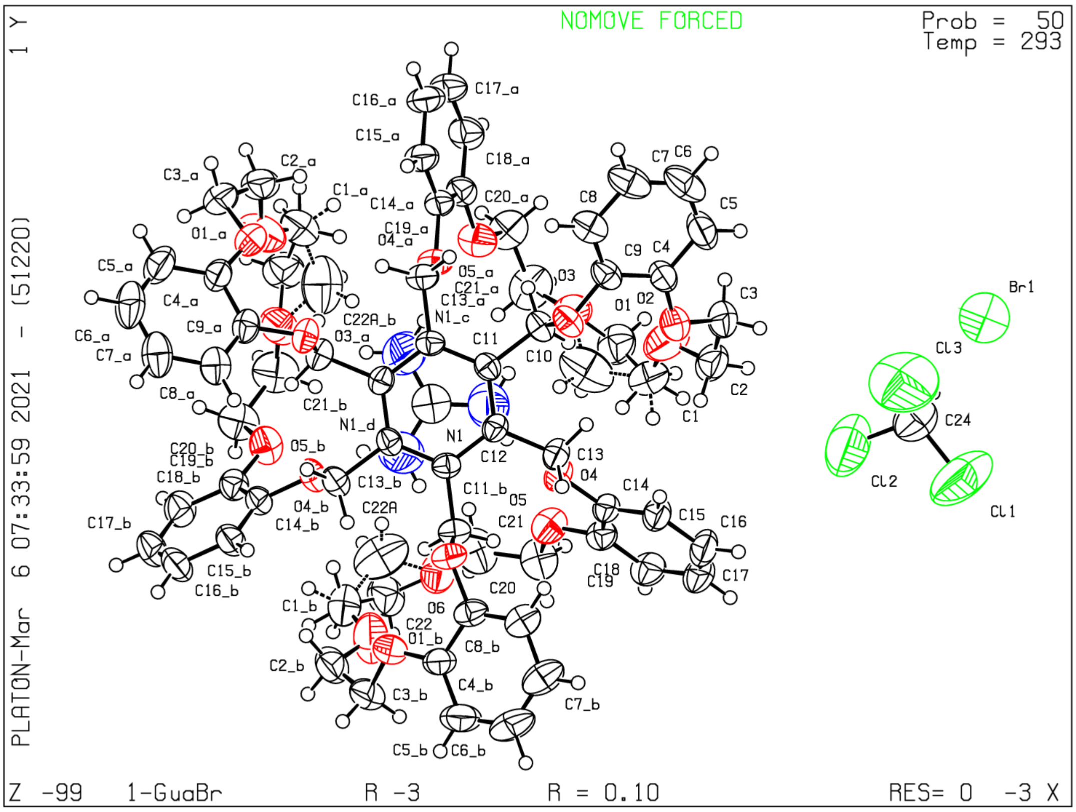

Fig. S44. View of complex $\mathbf{2}\cdot CN_3H_6^{+}\cdot CN_3H_5\cdot[Br(CHCl_3)_6]^{-}$. Displacement ellipsoids are scaled to the 50% probability level.

Table S3 Crystal data and structure refinement for 2-guanidium bromide.

Identification code	2-guanidium bromide
Empirical formula	$C_{140}H_{161}BrCl_{18}N_6O_{36}$
Formula weight	3221.75
Temperature/K	293(2)
Crystal system	trigonal
Space group	R-3
a/Å	21.8600(8)
b/Å	21.8600(8)
c/Å	27.7568(10)
α/°	90
β/°	90
γ/°	120
Volume/Å^3	11486.8(9)
Z	3
ρ_{calc}g/cm^3	1.397
μ/mm^{-1}	3.886
F(000)	5016.0
Crystal size/mm^3	0.125 × 0.114 × 0.086
Radiation	Cu Kα (λ = 1.54184 Å)
2Θ range for data collection/°	5.65 to 144.656
Index ranges	$-26 \le h \le 26$, $-24 \le k \le 21$, $-27 \le l \le 33$
Reflections collected	8089
Independent reflections	4902 [R_{int} = 0.0278, R_{sigma} = 0.0465]
Data/restraints/parameters	4902/1/313
Goodness-of-fit on F^2	1.068
Final R indexes [I>=2σ (I)]	R_1 = 0.1013, wR_2 = 0.2969
Final R indexes [all data]	R_1 = 0.1225, wR_2 = 0.3200
Largest diff. peak/hole / e Å^{-3}	0.52/-0.81
CCDC number	2068694

X-ray experimental for the complex 2-guanidium iodide

Single crystals of guanidium iodide complex of **2** were obtained as colorless cubes via the slow evaporation of a $CHCl_3$/CH_3OH solution of receptor **2** in the presence of excess guanidium iodide salt. A suitable crystal was selected and the data were collected on a Brucker D8 Advance diffractometer using a μ–focused Mo Kα radiation source (λ = 0.71073 Å). The crystal was kept at 150 K during data collection. Using Olex2,[10] the structure was solved with the ShelXT[11] structure solution program using Direct Methods and refined with the ShelXL[12] refinement package using Least Squares minimization. Tables of positional and thermal parameters, bond lengths and angles, torsion angles and figures are in the CIF file. CCDC deposition number: 2068695.

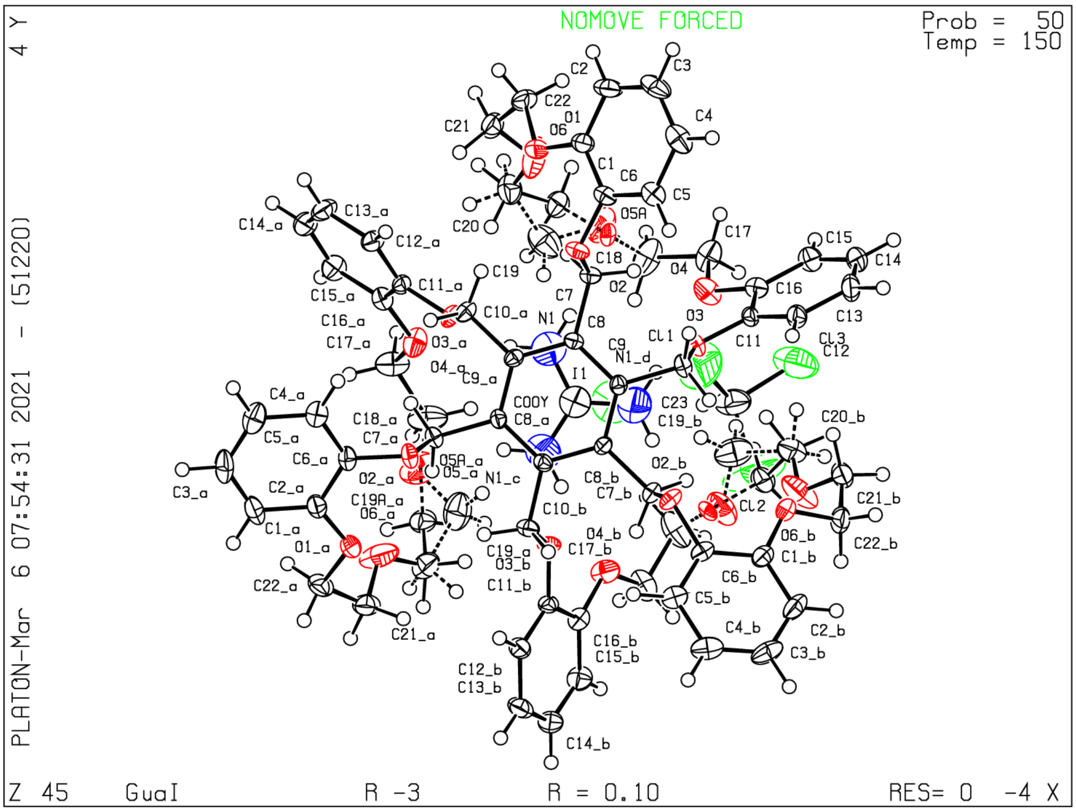

Fig. S45. View of complex **2**·$CN_3H_6^+$·CN_3H_5·$[I(CHCl_3)_6]^-$.. Displacement ellipsoids are scaled to the 50% probability level.

Table S4 Crystal data and structure refinement for 2-guanidium iodide.

Identification code	2-guanidium iodide
Empirical formula	$C_{140}H_{161}Cl_{18}IN_6O_{36}$
Formula weight	3268.74
Temperature/K	150.15
Crystal system	trigonal
Space group	R-3
a/Å	21.738(3)
b/Å	21.738(3)
c/Å	27.712(5)
α/°	90
β/°	90
γ/°	120
Volume/Å^3	11341(4)
Z	3
ρ_{calc}g/cm^3	1.436
μ/mm^{-1}	0.607
F(000)	5070.0
Crystal size/mm^3	0.34 × 0.332 × 0.315
Radiation	MoKα (λ = 0.71073 Å)
2Θ range for data collection/°	4.41 to 56.59
Index ranges	$-27 \leq h \leq 28, -28 \leq k \leq 28, -31 \leq l \leq 36$
Reflections collected	40495
Independent reflections	6249 [R_{int} = 0.0473, R_{sigma} = 0.0324]
Data/restraints/parameters	6249/2/323
Goodness-of-fit on F^2	1.113
Final R indexes [I>=2σ (I)]	R_1 = 0.1032, wR_2 = 0.2752
Final R indexes [all data]	R_1 = 0.1106, wR_2 = 0.2797
Largest diff. peak/hole / e Å^{-3}	1.21/-1.82
CCDC	2068695

X-ray experimental for the complex 2-guanidium bromide ($CHBr_3$)

Single crystals of guanidium bromide complex of **2** were obtained as colorless cubes via the slow evaporation of a $CHBr_3/CH_3OH$ solution of receptor **2** in the presence of excess guanidium chloride. A suitable crystal was selected and the data were collected on an Agilent Technologies SuperNova Dual Source diffractometer using a μ-focus CuKα radiation source (λ = 1.5418 Å) with collimating mirror monochromators. The crystal was kept at 100.15 K during data collection. The crystal was kept at 150 K during data collection. Using Olex2,[10] the structure was solved with the ShelXT[11] structure solution program using Direct Methods and refined with the ShelXL[12] refinement package using Least Squares minimization. Tables of positional and thermal parameters, bond lengths and angles, torsion angles and figures are in the CIF file. CCDC deposition number: 2088519.

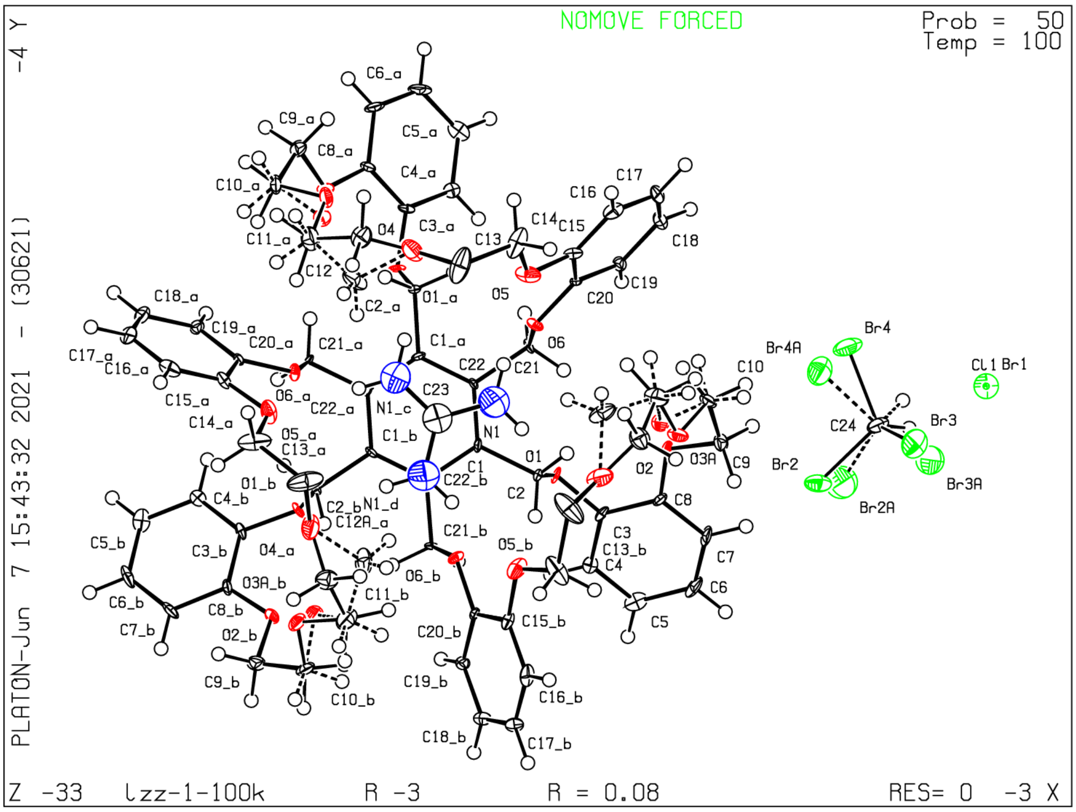

Fig. S46. View of complex $\mathbf{2}\cdot CN_3H_6^+\cdot CN_3H_5\cdot[Br(CHBr_3)_6]^-$. Displacement ellipsoids are scaled to the 50% probability level.

Table S5 Crystal data and structure refinement for 2-guanidium bromide ($CHBr_3$).

Empirical formula	$C_{140}H_{150}Br_{18.56}Cl_{0.44}N_6O_{36}$
Formula weight	3991.23
Temperature/K	100.0(4)
Crystal system	trigonal
Space group	R-3
a/Å	21.83560(10)
b/Å	21.83560(10)
c/Å	27.6959(2)
α/°	90
β/°	90
γ/°	120
Volume/Å^3	11436.05(13)
Z	3
ρ_{calc}g/cm^3	1.739
μ/mm^{-1}	6.464
F(000)	5931.0
Crystal size/mm^3	0.102 × 0.084 × 0.065
Radiation	Cu Kα (λ = 1.54184 Å)
2Θ range for data collection/°	5.658 to 151.72
Index ranges	$-27 \leq h \leq 27$, $-26 \leq k \leq 23$, $-34 \leq l \leq 34$
Reflections collected	23902
Independent reflections	5245 [R_{int} = 0.0464, R_{sigma} = 0.0235]
Data/restraints/parameters	5245/33/354
Goodness-of-fit on F^2	1.178
Final R indexes [I>=2σ (I)]	R_1 = 0.0848, wR_2 = 0.1964
Final R indexes [all data]	R_1 = 0.0851, wR_2 = 0.1965
Largest diff. peak/hole / e Å^{-3}	1.76/-1.73
CCDC number	2088519

6. HRMS spectra and NMR spectra

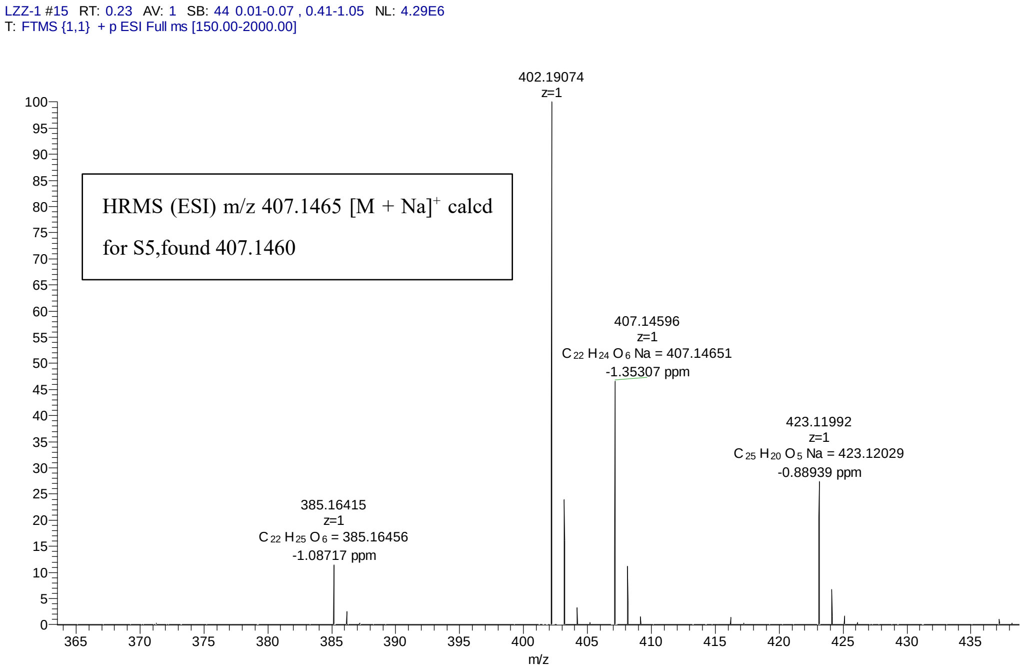

Fig. 47. HRMS spectrum of compound **S4**.

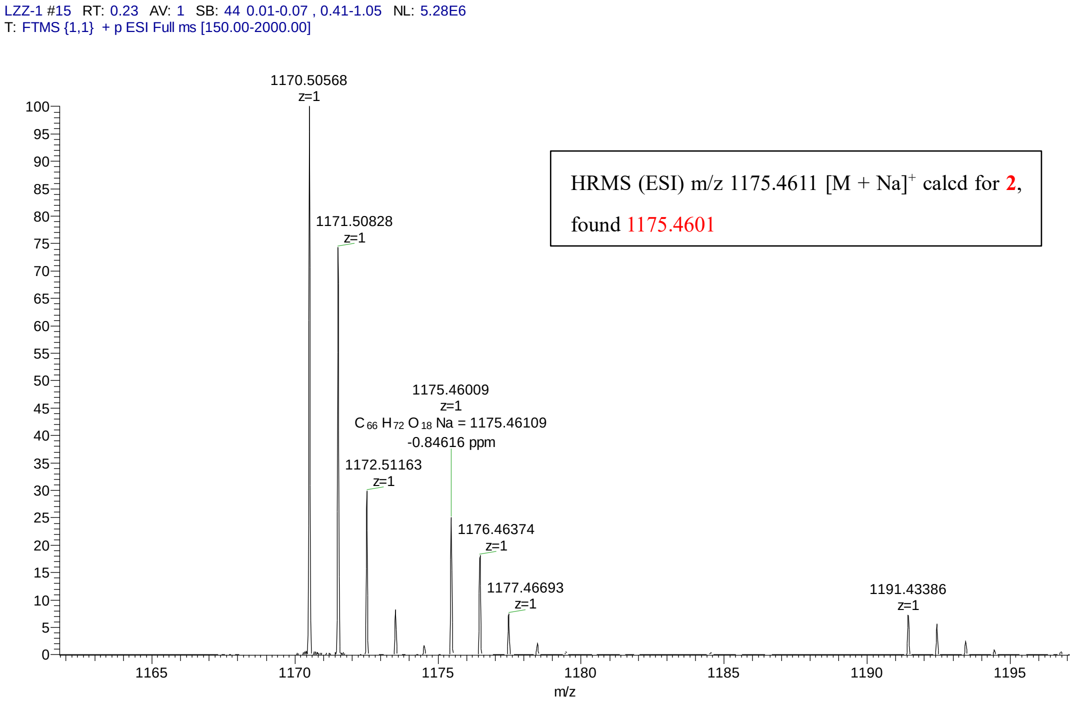

Fig. 48. HRMS spectrum of compound **2**.

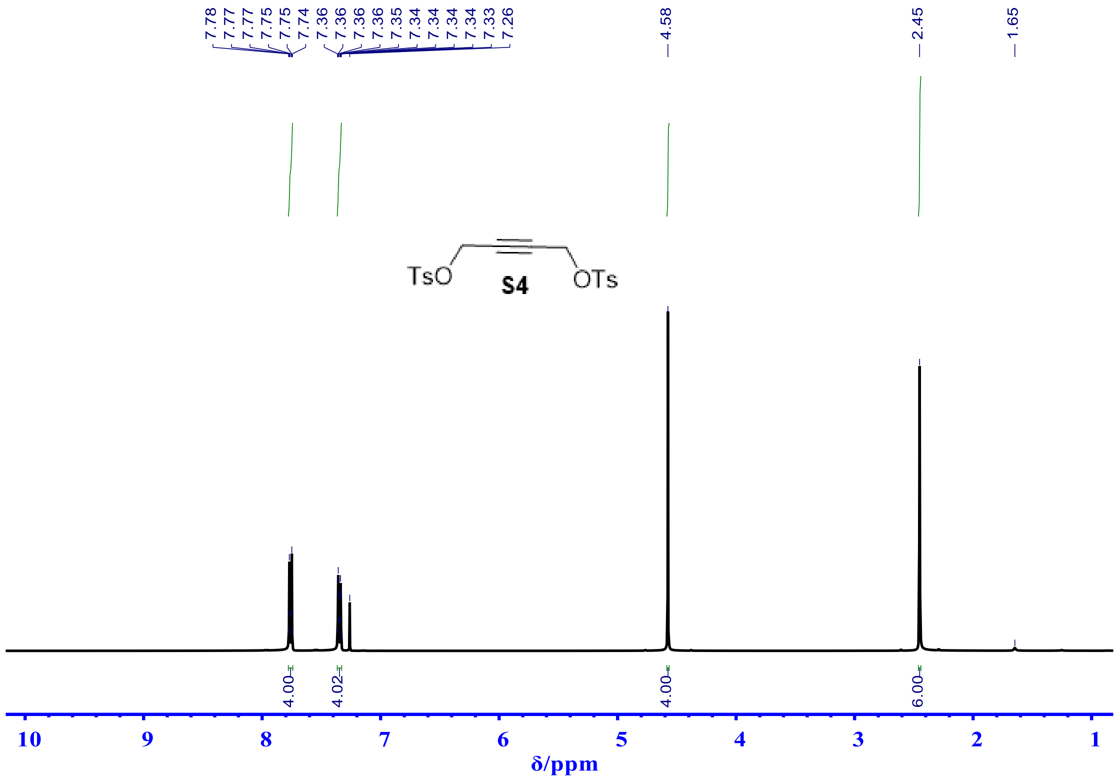

Fig. S49 ^{1}H NMR spectrum of **S4** recorded in $CDCl_3$.

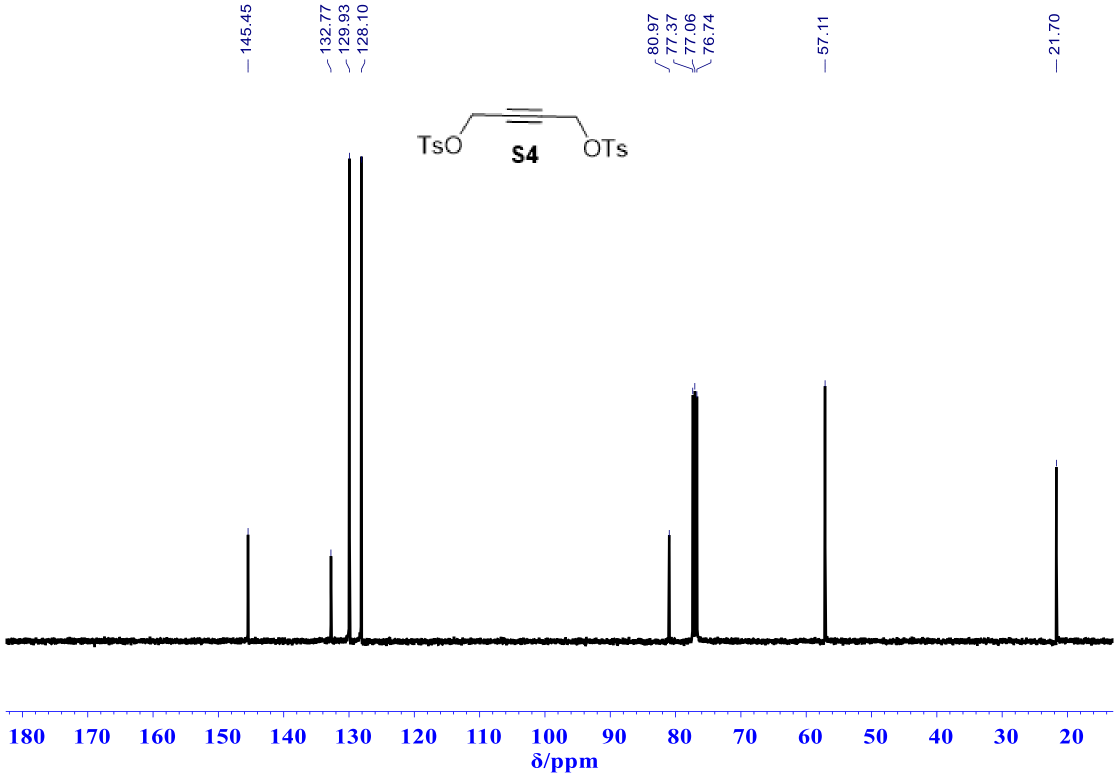

Fig. S50 ^{13}C NMR spectrum of **S4** recorded in $CDCl_3$.

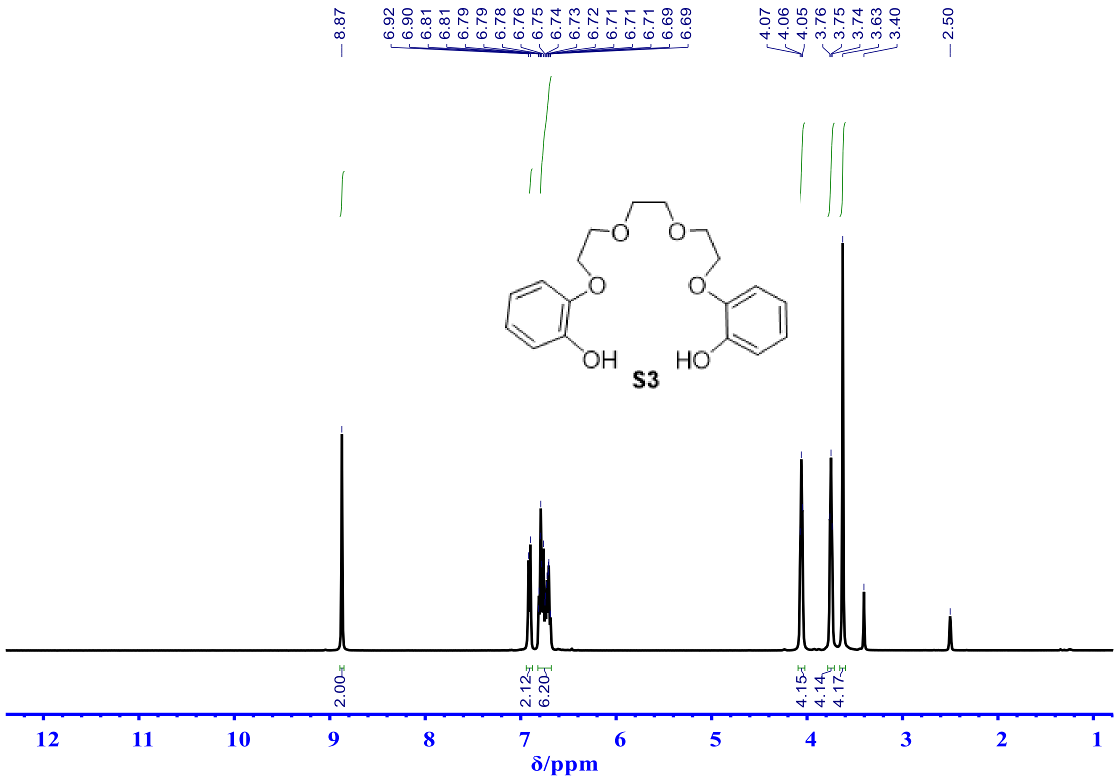

Fig. S51 ¹H NMR spectrum of **S3** recorded in DMSO-d6.

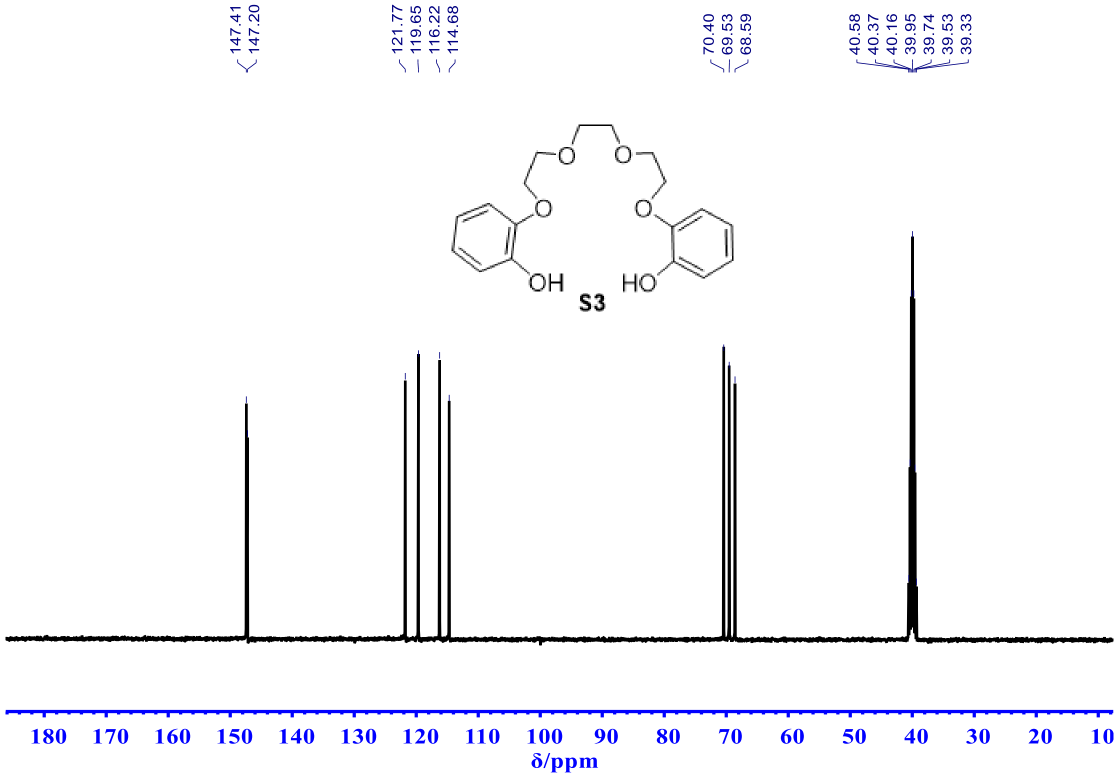

Fig. S52 ¹³C NMR spectrum of **S3** recorded in DMSO-d6.

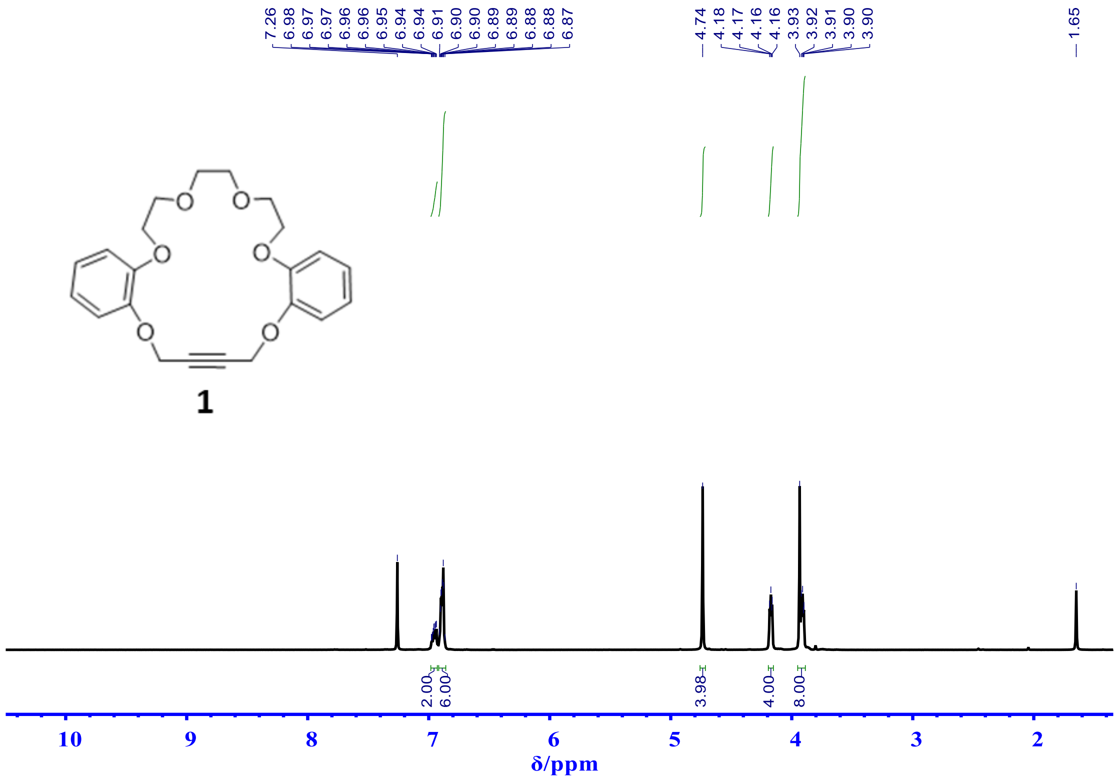

Fig. S53 ^{1}H NMR spectrum of **1** recorded in $CDCl_3$

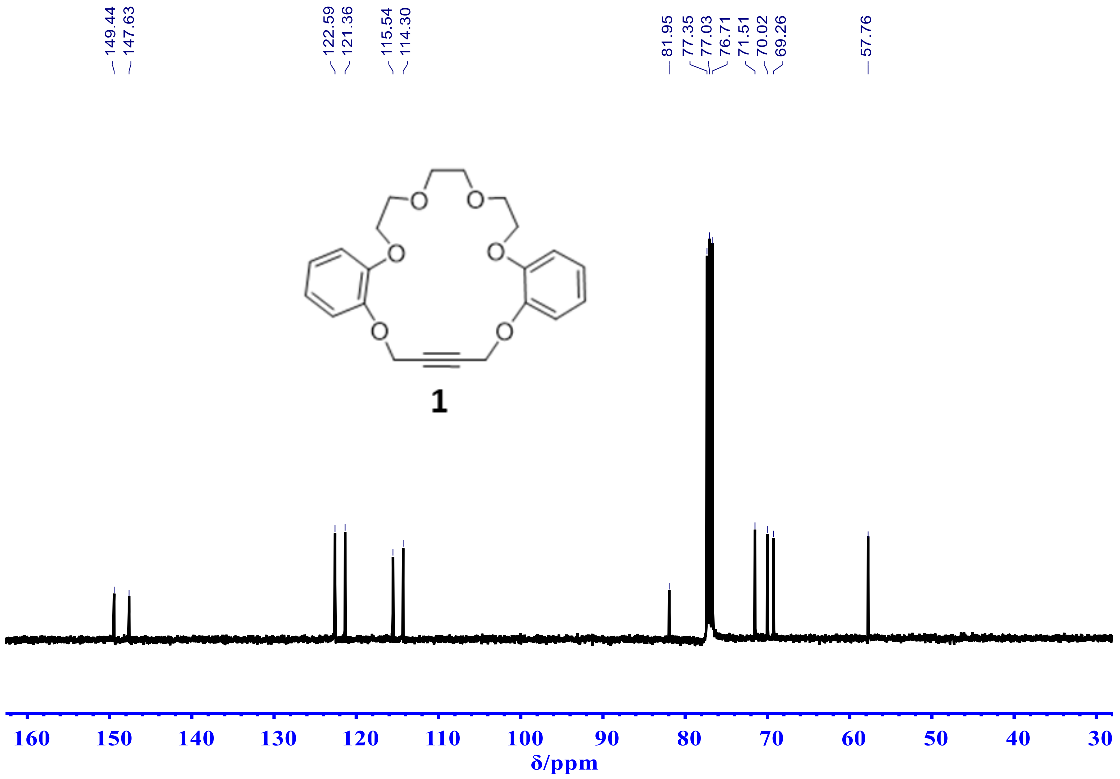

Fig. S54 ^{13}C NMR spectrum of **1** recorded in $CDCl_3$

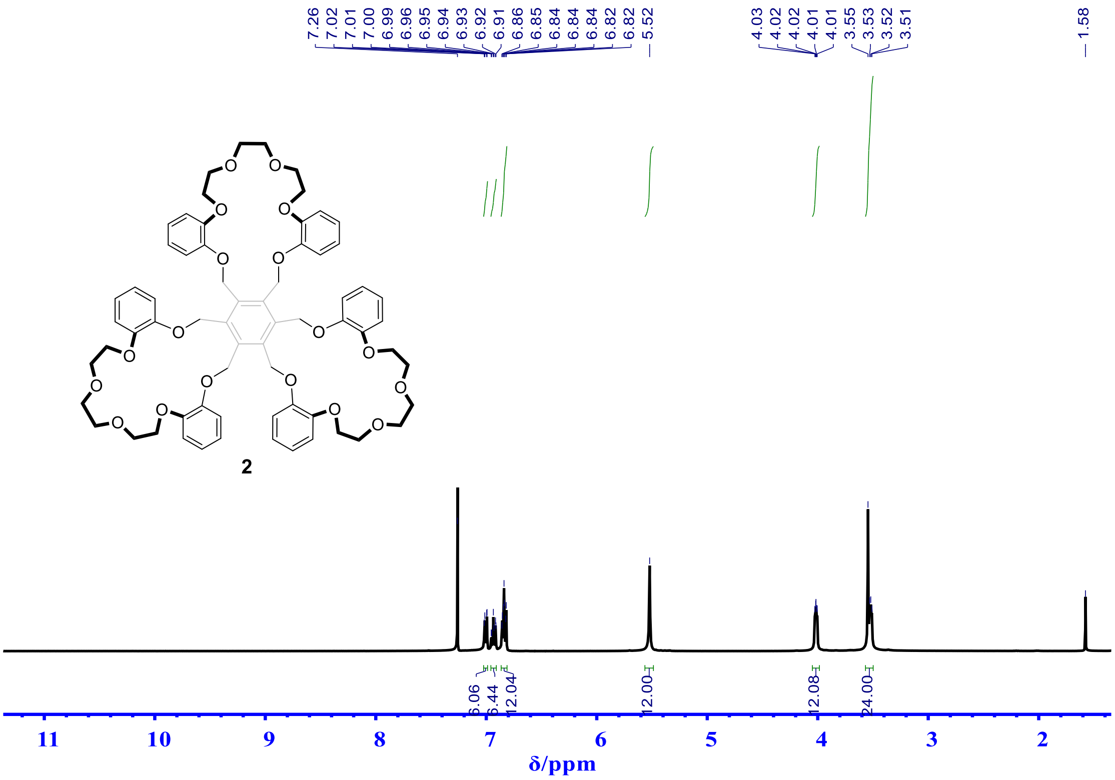

Fig. S55 ^{1}H NMR spectrum of **2** recorded in $CDCl_3$

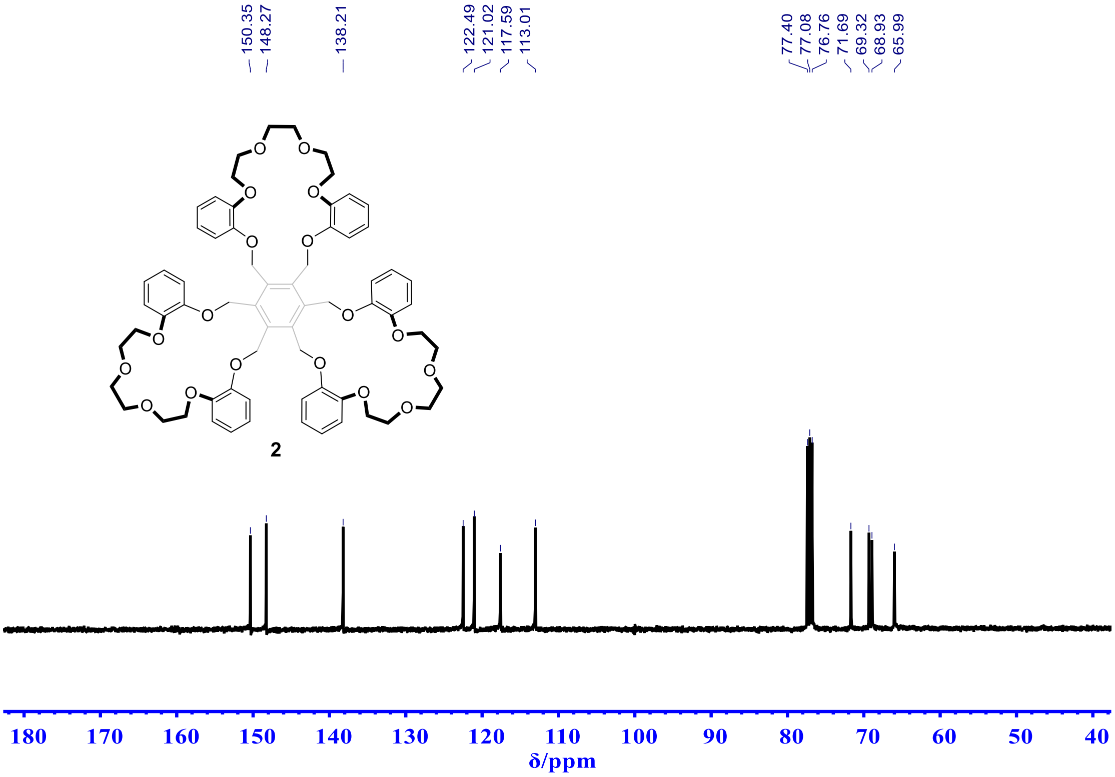

Fig. S56 ^{13}C NMR spectrum of **2** recorded in $CDCl_3$

7. REFERENCES

1. Gaussian 09, R. E., Frisch, M. J.; Trucks, G. W.; Schlegel, H. B.; Scuseria, G. E.; Robb, M. A.; Cheeseman, J. R.; Scalmani, G.; Barone, V.; Mennucci, B.; Petersson, G. A.; Nakatsuji, H.; Caricato, M.; Li, X.; Hratchian, H. P.; Izmaylov, A. F.; Bloino, J.; Zheng, G.; Sonnenberg, J. L.; Hada, M.; Ehara, M.; Toyota, K.; Fukuda, R.; Hasegawa, J.; Ishida, M.; Nakajima, T.; Honda, Y.; Kitao, O.; Nakai, H.; Vreven, T.; Montgomery, Jr., J. A.; Peralta, J. E.; Ogliaro, F.; Bearpark, M.; Heyd, J. J.; Brothers, E.; Kudin, K. N.; Staroverov, V. N.; Kobayashi, R.; Normand, J.; Raghavachari, K.; Rendell, A.; Burant, J. C.; Iyengar, S. S.; Tomasi, J.; Cossi, M.; Rega, N.; Millam, J. M.; Klene, M.; Knox, J. E.; Cross, J. B.; Bakken, V.; Adamo, C.; Jaramillo, J.; Gomperts, R.; Stratmann, R. E.; Yazyev, O.; Austin, A. J.; Cammi, R.; Pomelli, C.; Ochterski, J. W.; Martin, R. L.; Morokuma, K.; Zakrzewski, V. G.; Voth, G. A.; Salvador, P.; Dannenberg, J. J.; Dapprich, S.; Daniels, A. D.; Farkas, Ö.; Foresman, J. B.; Ortiz, J. V.; Cioslowski, J.; and Fox, D. J., Gaussian, Inc., Wallingford CT, 2009.
2. Xu, X.; Goddard, W. A., The X3LYP extended density functional for accurate descriptions of nonbond interactions, spin states, and thermochemical properties. *P Natl Acad Sci USA* **2004,** *101* (9), 2673-2677.
3. Boys, S. F.; Bernardi, F., The calculation of small molecular interactions by the differences of separate total energies. Some procedures with reduced errors (Reprinted from Molecular Physics, vol 19, pg 553-566, 1970). *Mol Phys* **2002,** *100* (1), 65-73.
4. van Duijneveldt, F. B.; van Duijneveldt-van de Rijdt, J. G. C. M.; van Lenthe, J. H., State of the Art in Counterpoise Theory. *Chem Rev* **1994,** *94* (7), 1873-1885.
5. Kyba, E. P.; Helgeson, R. C.; Madan, K.; Gokel, G. W.; Tarnowski, T. L.; Moore, S. S.; Cram, D. J., Host-guest complexation. 1. Concept and illustration. *J Am Chem Soc* **1977,** *99* (8), 2564-2571.
6. Geiger, T.; Haupt, A.; Maichle-Mossmer, C.; Schrenk, C.; Schnepf, A.; Bettinger, H. F., Synthesis and Photodimerization of 2-and 2,3-Disubstituted Anthracenes: Influence of Steric Interactions and London Dispersion on Diastereoselectivity. *J Org Chem* **2019,** *84* (16), 10120-10135.
7. Kotha, S.; Waghule, G. T., Diversity Oriented Approach to Crownophanes by Enyne Metathesis and Diels-Alder Reaction as Key Steps. *J Org Chem* **2012,** *77* (14), 6314-6318.
8. Spek, A. L., PLATON SQUEEZE: a tool for the calculation of the disordered solvent contribution to the calculated structure factors. *Acta Crystallographica Section C-Structural Chemistry* **2015,** *71*, 9-18.
9. BindFit. www.supramolecular.org (accessed February 26.
10. Dolomanov, O. V.; Bourhis, L. J.; Gildea, R. J.; Howard, J. A. K.; Puschmann, H., OLEX2: a complete structure solution, refinement and analysis program. *J Appl Crystallogr* **2009,** *42*, 339-341.
11. Sheldrick, G. M., SHELXT - Integrated space-group and crystal-structure determination. *Acta Crystallogr A* **2015,** *71*, 3-8.
12. Sheldrick, G. M., Crystal structure refinement with SHELXL. *Acta Crystallogr C* **2015,** *71*, 3-8.
